# Enantioselective C–H Activations by Metallaelectro-Catalysis

**DOI:** 10.1021/acs.accounts.6c00509

**Published:** 2026-09-23

**Authors:** Sven Erik Peters, Sven Trienes, Yanjun Li, Lutz Ackermann

**Affiliations:** † Wöhler Research Institute for Sustainable Chemistry (WISCh), 9375 Georg-August-Universität Göttingen, Göttingen, Lower Saxony 37077, Germany; ‡ Clinic for General, Visceral and Paediatric Surgery, University Medical Center Göttingen, Georg-August-Universität Göttingen, Göttingen, Lower Saxony 37075, Germany

## Abstract

Given the increasing awareness of the economic and environmental aspects of chemical syntheses, transformative catalysis platforms have emerged, with molecular electrocatalysis residing at the forefront. It enables sustainable oxidative transformations with high levels of energy efficiency and resource economy as well as scalability and inherent process safety. Hence, organic electrocatalysis has gained considerable momentum. Further, in terms of sustainable syntheses, the catalyzed activation of otherwise inert C–H bonds was identified as a particularly powerful strategy for the rapid assembly of chiral scaffolds, thereby preventing stoichiometric chemical waste. Despite indisputable advances, these strategies largely relied, until recently, on stoichiometric quantities of often expensive or toxic chemical oxidants. To this end, metallaelectro-catalysis has emerged as an enabling technology for C–H activations, using the traceless transfer of protons and electrons *in lieu* of chemical oxidants, while generating molecular hydrogen as the sole by-product *via* the synchronized hydrogen evolution reaction (HER). Although distinct electrooxidative C–H activations were realized during the past decade, enantioselective electrochemical catalysis has thus far proven elusive. This Account summarizes our key advances until August 2026 in metallaelectro-catalyzed enantioselective C–H activation, streamlining the assembly of chiral molecules featuring various stereogenic elements. Thus, palladaelectro-catalyzed C–H alkenylation set the stage for oxidative C–H/C–H couplings toward atropochiral biaryls, leveraging chiral transient directing groups (TDGs). Subsequent research efforts have allowed us to broaden this platform. Since the pronounced complexity of the electrochemical space in these systems can complicate discovery and optimization, the implementation of a machine learning (ML) workflow was targeted. This was harnessed for the accelerated optimization of an enantioselective palladaelectro-catalyzed annulation reaction. Moreover, the versatility of rhodium catalysis in enantioselective electrochemical C–H activations was mirrored by the e construction of chiral spiropyrazolones and phthalides. Tailored ruthenium catalysts allowed for the selective buildup of atropostable indoles as well as chiral spirocyclic compounds. Taking into consideration the environmental implications and cost-efficacy of transition metal-catalyzed transformations, we further explored the utility of earth-abundant transition metals. On this note, enantioselective cobaltaelectro-catalysis enabled a broad range of highly efficient and enantioselective transformations, such as spirocyclizations, annulations with alkynes, allenes, and alkenes toward axial-, *C*-, and *P*-centered chirality, as well as decagram-scale synthesis realized in flow. The data-driven exploration of newly designed chiral ligands facilitated accelerated discovery in cobaltaelectro-catalyzed C–H activation. Moreover, cupraelectro-catalysis was employed for crafting planar chirality in ferrocene derivatives through versatile C–Het bond formations. Finally, we achieved an enantioselective desymmetrization through electrooxidative nickel catalysis inspired by ML, selectively constructing multiple contiguous stereocenters. Interestingly, comparative analysis revealed distinct reactivity for cobalt and nickel catalysts, whose mechanistic origins were elucidated.

## Graphical abstract

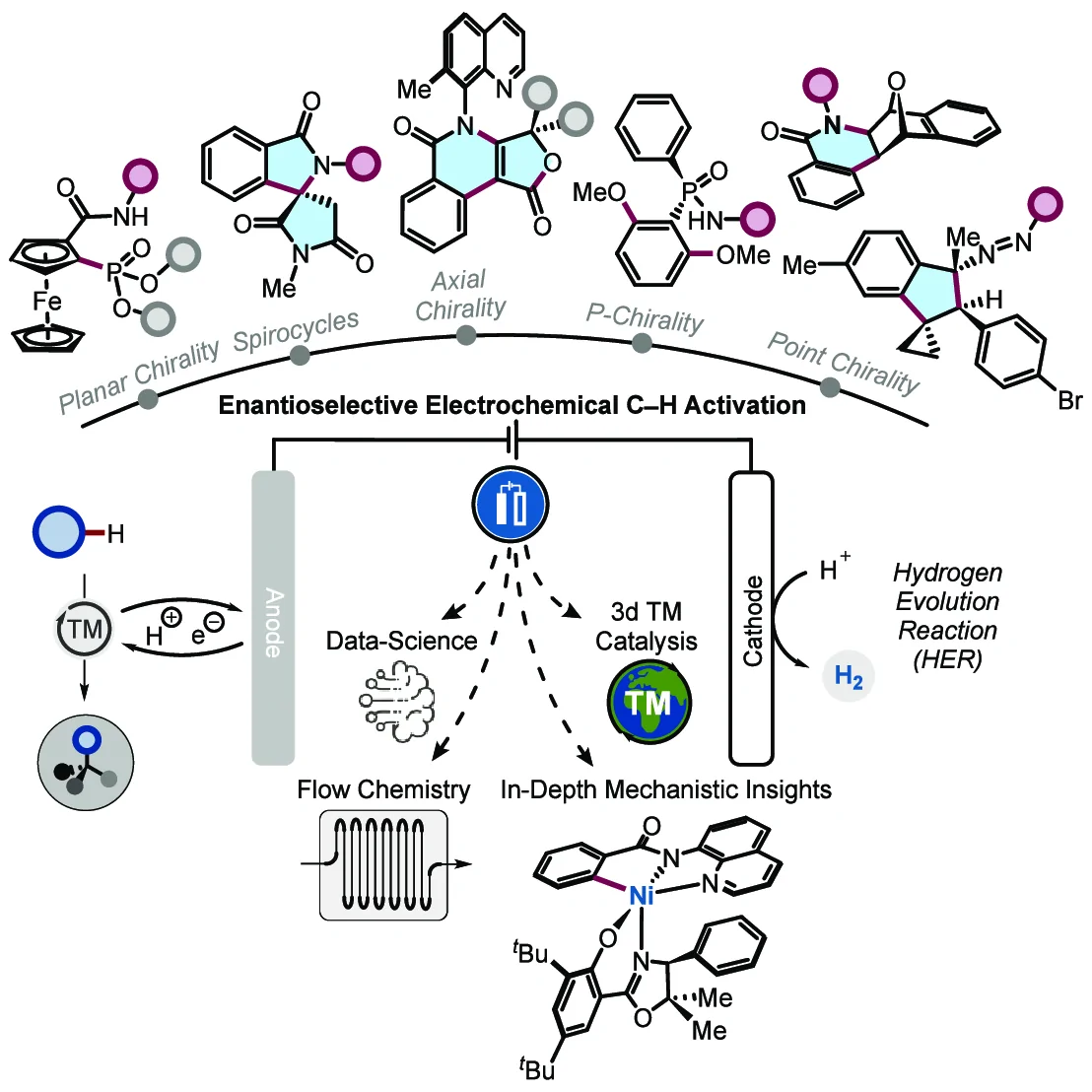

## Key References

-

Dhawa, U. ; Tian, C. ; Wdowik, T. ; Oliveira, J. C. A. ; Hao, J. ; Ackermann, L. Enantioselective Pallada-Electrocatalyzed C–H Activation by Transient Directing Groups: Expedient Access to Helicenes. Angew. Chem. Int. Ed. 2020, 59, 13451–13457.10.1002/anie.202003826PMC749711632243685 ^1^ This work is the first example of metallaelectro-catalyzed enantioselective C–H activation while merging palladium catalysis and a chiral transient directing group.
-

Hou, X. ; Li, S. ; Frey, J. ; Hong, X. ; Ackermann, L. Machine Learning-Guided Yield Optimization for Palladaelectro-catalyzed Annulation Reaction. Chem 2024, 10, 2283–2294.^2^ This work introduces a ML-guided workflow for the directed optimization of enantioselective palladaelectro-catalyzed annulation in a high-dimensional chemical space.
-

von Münchow, T. ; Dana, S. ; Xu, Y. ; Yuan, B. ; Ackermann, L. Enantioselective electrochemical cobalt-catalyzed aryl C–H activation reactions. Science 2023, 379, 1036–1042.10.1126/science.adg286636893225 ^3^ This study disclosed an unprecedented merger of enantioselective cobalt-catalyzed C–H activation and electrocatalysis, demonstrating the viability of 3d transition metal catalysis in a broad array of transformations.
-

von Münchow, T. ; Pandit, N. K. ; Dana, S. ; Boos, P. ; Peters, S. E. ; Boucat, J. ; Liu, Y.-R. ; Scheremetjew, A. ; Ackermann, L. Enantioselective C–H annulations enabled by either nickel- or cobalt-electrocatalysed C–H activation for catalyst-controlled chemodivergence. Nat. Catal. 2025, 8, 257–269.^4^ This study introduced enantioselective nickelaelectro-catalyzed C–H activation and showcased the beneficial potential of interdisciplinary efforts through a ML-guided ligand optimization.

## 1 Introduction

Electrochemistry has emerged as a transformative platform for sustainable molecular synthesis, featuring a unique potential for resource-economical transformations.^5–11^ Thus, molecular electrochemistry underwent a remarkable renaissance as an enabling interdisciplinary toolbox. The biggest impact is arguably on redox transformations, which can be accomplished under exceedingly mild conditions in the absence of stoichiometric chemical redox reagents, thereby enabling waste reduction and process safety in an economically attractive fashion (Scheme 1A). Thus, the flow of protons and electrons can replace chemical oxidants and sets the stage for unique reaction pathways through single electron transfers (SET). Additionally, tailored selectivities can be dialed in by the precise control of the applied potential.^12,13^ As a result, molecular electrochemistry has witnessed a strong demand in both industry and academia toward, among others, implementation on scale as well as the assembly of structurally complex molecules.^14–17^

**Scheme 1:**
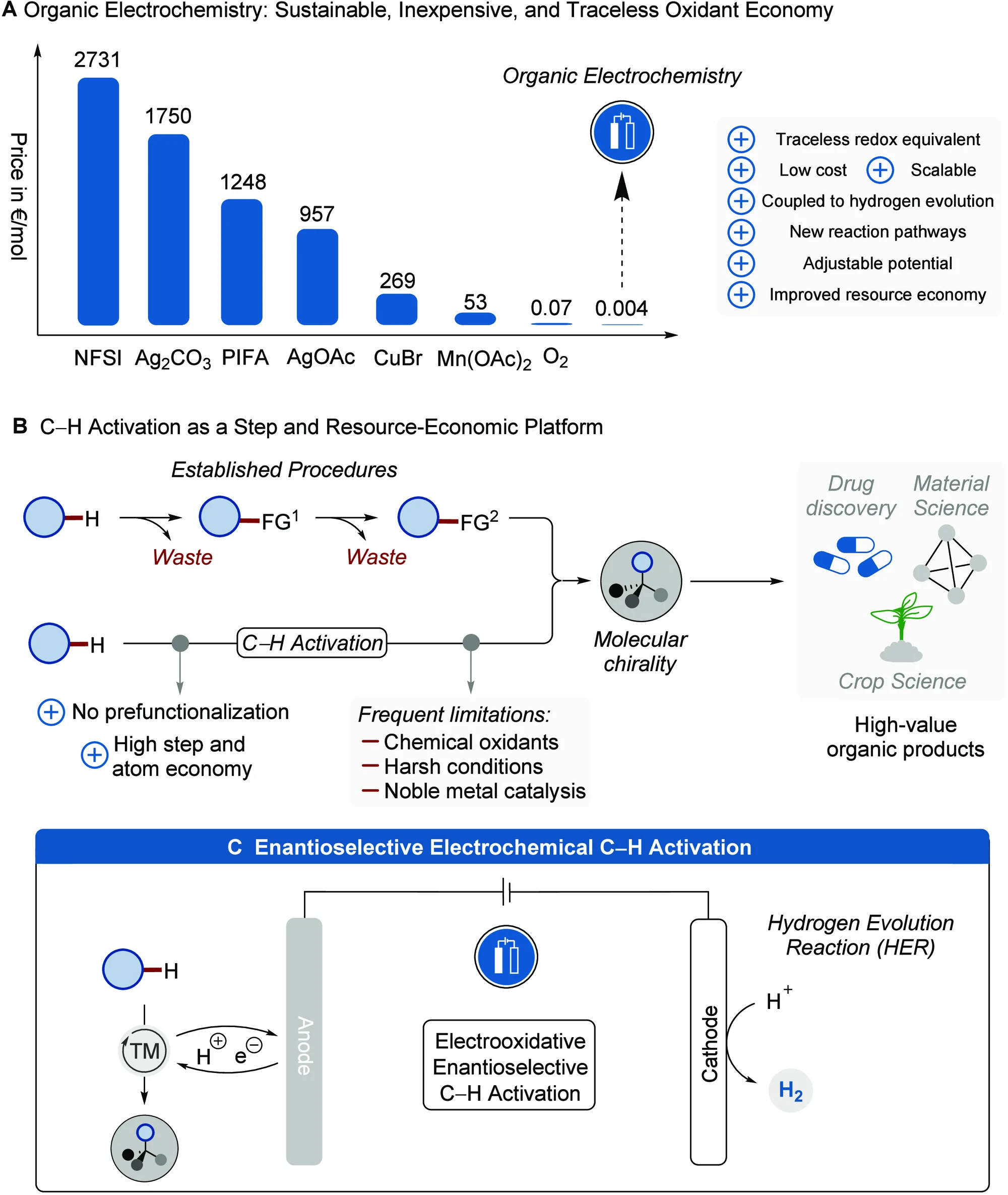
A) Cost Comparison for Commonly Used Oxidants; B) C–H Activation as a Resource-Economical Strategy; C) Enantioselective Metallaelectro-Catalyzed C–H Activation^a^

a Average prices for the largest units obtained from abcr, Fisher Scientific, Sigma-Aldrich, and Nippon-Gases.

Transition metal-catalyzed C–H activation has surfaced as an exceptionally powerful strategy for an accelerated transition toward sustainable, cost-effective synthesis.^18,19^ Omnipresent C–H bonds can thus be reimagined as latent functional groups by means of direct functionalization, circumventing the need for waste-generating and energy-intensive prefunctionalization.^19–21^ Crucially, it represents a transformative tool for the assembly of chiral molecules (Scheme 1B).^22–24^ Despite recent progress, contemporary advances are largely limited by costly or toxic chemical oxidants, harsh reaction conditions, and high loadings of noble metals.^25–28^ In this context, the benefits of molecular electrochemistry provide a promising solution (Scheme 1C). Hence, the merger of C–H activation with electrochemistry within metallaelectro-catalysis has been identified as one of the most promising strategies for scalable electrooxidations.^29–32^

The electron transfer occurs either through direct heterogeneous transfer at the electrode with the transition metal complex or, in selected cases, with the aid of an additional redox mediator as electron shuttle. Thereby, electricity does not solely serve as a simple replacement of chemical oxidants. The generally milder conditions can beneficially influence stereoinduction and functional group tolerance. Further, the fine control of the externally applied potential opens the possibility to directly influence selectivity and catalyst speciation, while diminishing the influence of racemic background reactions.^33,34^ The SET-driven access to novel reaction pathways enables the practitioner to fully harness the redox flexibility of 3d transition metals, rendering them specifically attractive in this research area.^30^ In addition, oxidation-induced reductive elimination manifolds are often viable by means of electrocatalysis.^35–37^ Notably, the cathodic hydrogen evolution reaction (HER) through proton reduction is frequently harnessed for a closed electric circuit, intertwining the construction of molecular complexity with a potential future decentralized green hydrogen economy.^38,39^

Nevertheless, electrocatalysis is associated with diverse challenges toward efficient stereoinduction. Most notably, the controlled avoidance of cathodic deposition of active intermediates and the decomposition of chiral ligand or other relevant species can pose substantial difficulties. Additionally, unfavorable interactions in the enantiodetermining step with, for instance, the supporting electrolyte or the electrodes hamper streamlined reaction discovery. Further aspects involve kinetic limitations regarding efficient mass and electron transport and restrictions regarding the choice of solvent. Despite these hurdles, highly enantioselective metallaelectro-catalyzed transformations have indeed been realized in recent years.^1–4,33,34^

We commenced our program on enantioselective electrochemical C–H activation in 2018.^1^ Since then, a diverse array of transformations were developed, capitalizing on the tamable reactivity of noble metals and, more importantly, on the superior sustainability of 3d transition metal catalysis. The complexity of these conversions was understood by extensive mechanistic studies, providing insights into the current’s precise role. Additionally, the interdisciplinary nature of the research field was appreciated by implementation of machine-learning (ML) approaches, facilitating accelerated discovery by predictive power and in-depth mechanistic understanding. In this Account, we summarize our findings on metallaelectro-catalyzed C–H activations until August 2026.

## 2 Noble Metallaelectro-Catalysis

The coordination chemistry of noble metals is exceptionally robust and well-investigated, rendering them ideal candidates for: (i) achieving efficient enantioinduction under electrochemical conditions despite the diverse challenges (*vide supra*) and (ii) accessing synthetically challenging transformations, such as assembling non-conventional but increasingly relevant stereogenic elements (e.g., chiral axis) or complex C­(sp^3^)-rich structures.^40–42^

Palladium ranks among the most powerful transition metals for selective C–H activation with early contributions by Fujiwara and Moritani as well as Ames and Ohta for palladium­(II/0) and palladium­(0/II) catalysis, respectively.^41–442^ Particularly, the construction of chiral architectures has strongly benefited from the power of chiral palladium complexes.^40,41,44,45^ However, previous strategies largely suffered from the use of stoichiometric chemical oxidants and the by-products derived thereof.^46,47^

In sharp contrast, and within our long-standing research interest in selective electrocatalysis,^29,30,48,49^ in 2020, we established a versatile platform for the enantioselective palladaelectro-catalyzed C–H activation.^1^ The C–H alkenylation reaction of biaryls **1** utilized a transient directing group (TDG) strategy,^50,51^ which allowed us to elegantly access axially chiral biaryls **3** with an atropostable C–C bond (Scheme 2). l-*tert*-Leucine, as a TDG from the chiral pool, forms an imine with the substrate’s aldehyde moiety, setting the stage for efficient stereoinduction. On this note, it was shown by density functional theory (DFT) calculations that attractive noncovalent interactions (NCIs) between the amino acid’s *tert*-butyl substituent and the substrate’s naphthyl group as well as the arene π-system and palladium are of importance for selectivity control. Overall, the developed metallaelectro-catalysis relied on a user-friendly undivided cell setup under galvanostatic conditions and featured outstanding functional group tolerance and enantioselectivities, accessing a diverse set of axially chiral benzaldehyde derivatives **3** (Scheme 2A). Remarkably, the generality of this platform was mirrored by the atroposelective synthesis of chiral *N*-aryl pyrroles, maintaining high chemical and optical yields (Scheme 2B). Finally, the outstanding utility of the metallaelectro-catalysis strategy was demonstrated by late-stage diversifications toward the enantiopure helicene **4** (Scheme 2C).^52^ Similarly, the telescoped modifications allowed for the synthesis of chiral BINOLS and dicarboxylic acids with substantial potential in terms of ligand development.

**Scheme 2:**
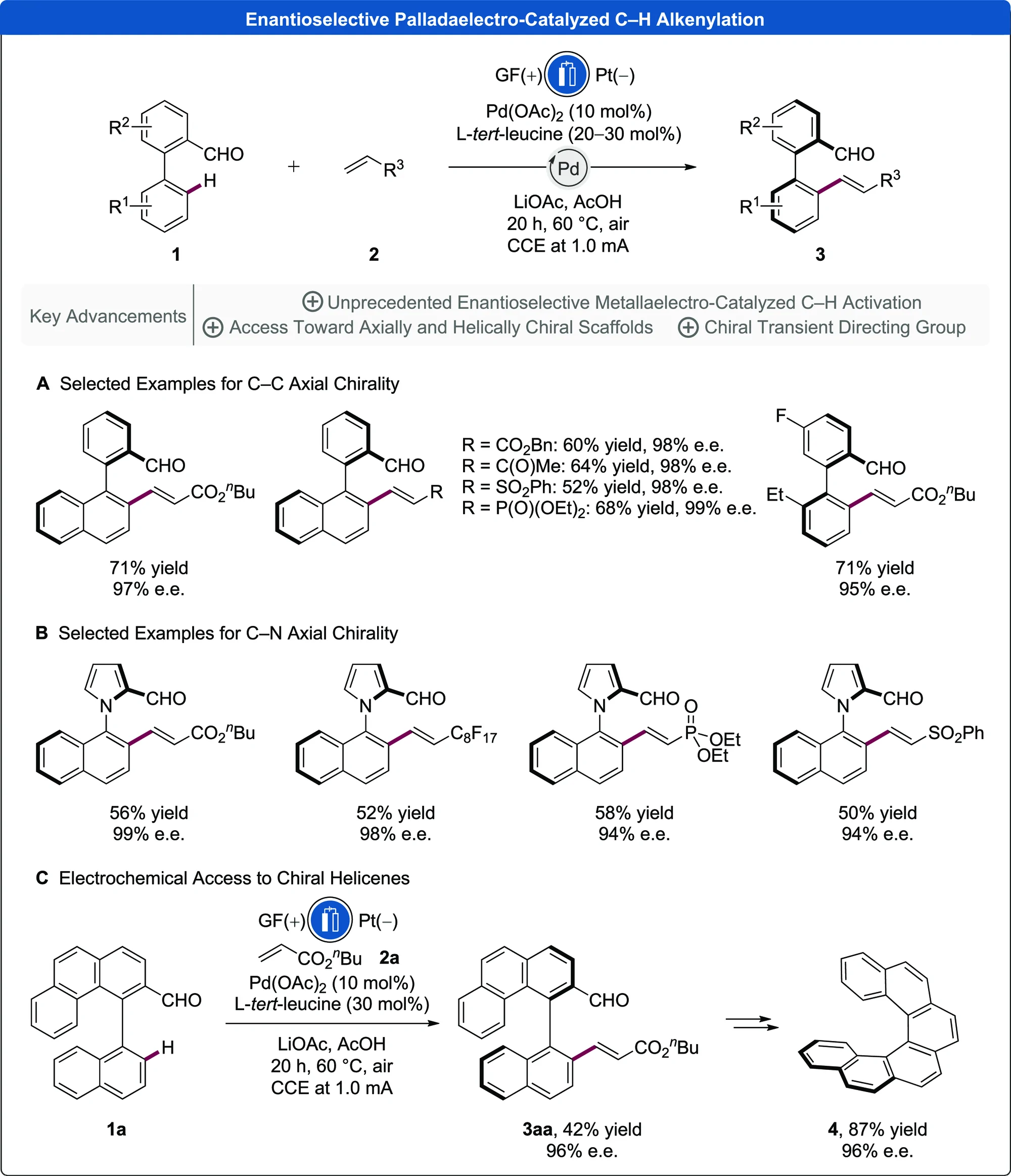
Atroposelective Synthesis of Axially Chiral Biaryls **3** Through Palladaelectro-Catalyzed C–H Alkenylation^a^

a GF = Graphite felt. CCE = Constant current electrolysis.

Heterobiaryls featuring C–N axial chirality are key structural motifs in natural products, pharmaceuticals, ligands, and catalysts.^53,54^ The enantioselective synthesis of such scaffolds continues to pose a significant challenge. Thus, our group disclosed an enantioselective palladaelectro-catalysis platform,^1^ offering an operationally simple, robust, and versatile approach to precisely tailor C–N atropostable heterobiaryls **8** and **9** (Scheme 3).^55^ The broad applicability of this strategy was reflected by a diverse set of alkenes **5**, containing ester, sulfonate, and phosphonate functionalities, affording the corresponding atropochiral products **8** with excellent enantioselectivities (Scheme 3A). Remarkably, maleimides, an essential motif in natural products and pharmaceuticals, were incorporated likewise with high levels of enantioinduction. Moreover, C–H allylation using 1,1-disubstituted alkenes **6** proved to be viable, furnishing allylated C–N axially chiral heterobiaryls **9** (Scheme 3B).

**Scheme 3:**
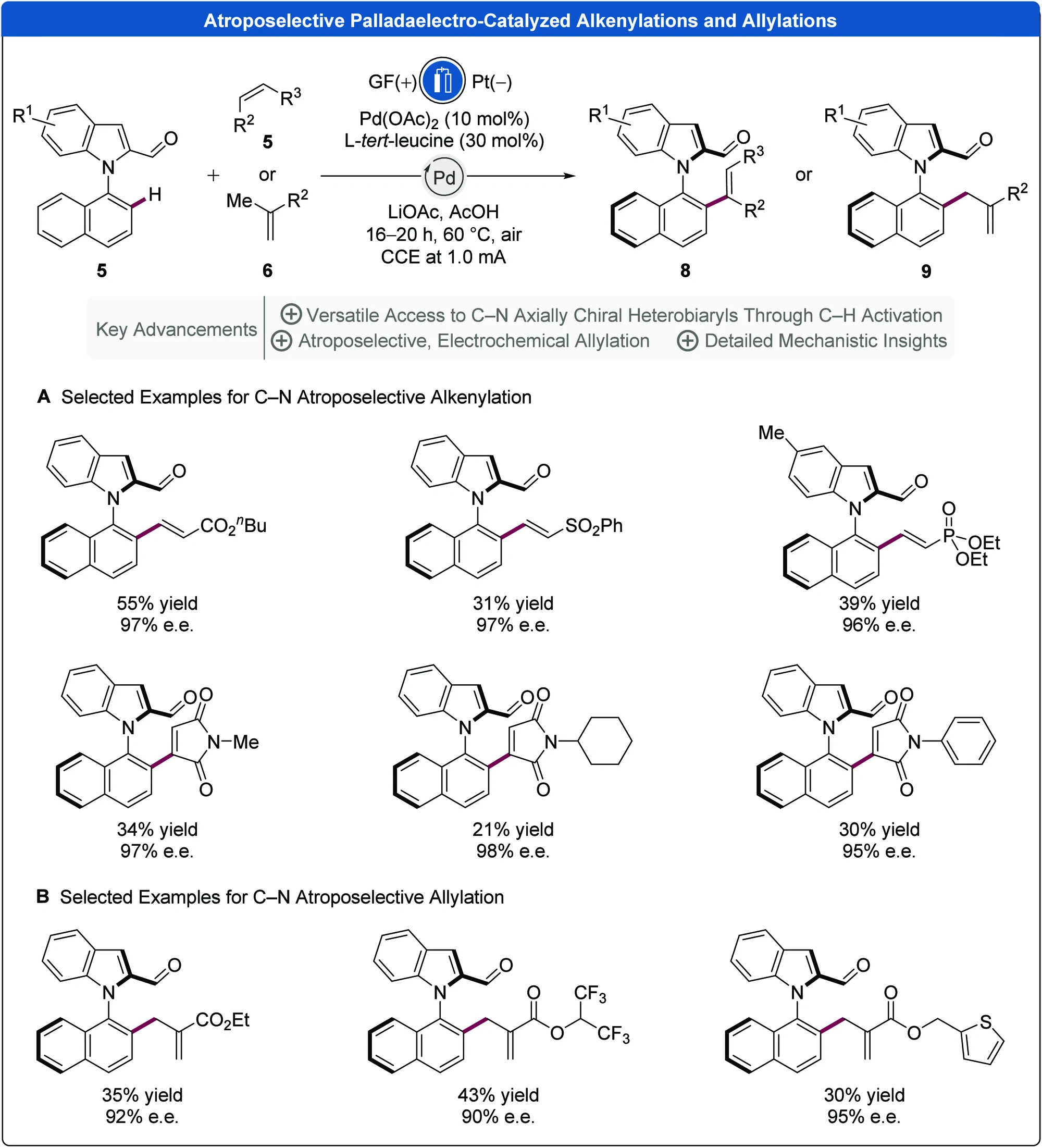
Enantioselective Palladaelectro-Catalyzed Alkenylation and Allylation Toward Heterobiaryls **8** and **9**

In order to understand the *modus operandi* of our palladaelectro-catalyzed enantioselective platforms, we performed detailed computational and electroanalytical investigations,^55^ which provided strong support for a proposed reaction mechanism (Scheme 4). Thereby, Pd complex **Pd1** is initially formed through the coordination of *in situ* generated imine, carrying the chiral TDG. Subsequent C–H activation and olefin coordination gives rise to species **Pd2**. Afterward, migratory insertion leads to the formation of the desired C–C bond (**Pd3**), which after β-H elimination provides access to the desired product. This is followed by the regeneration of the active catalyst through anodic oxidation.

**Scheme 4:**
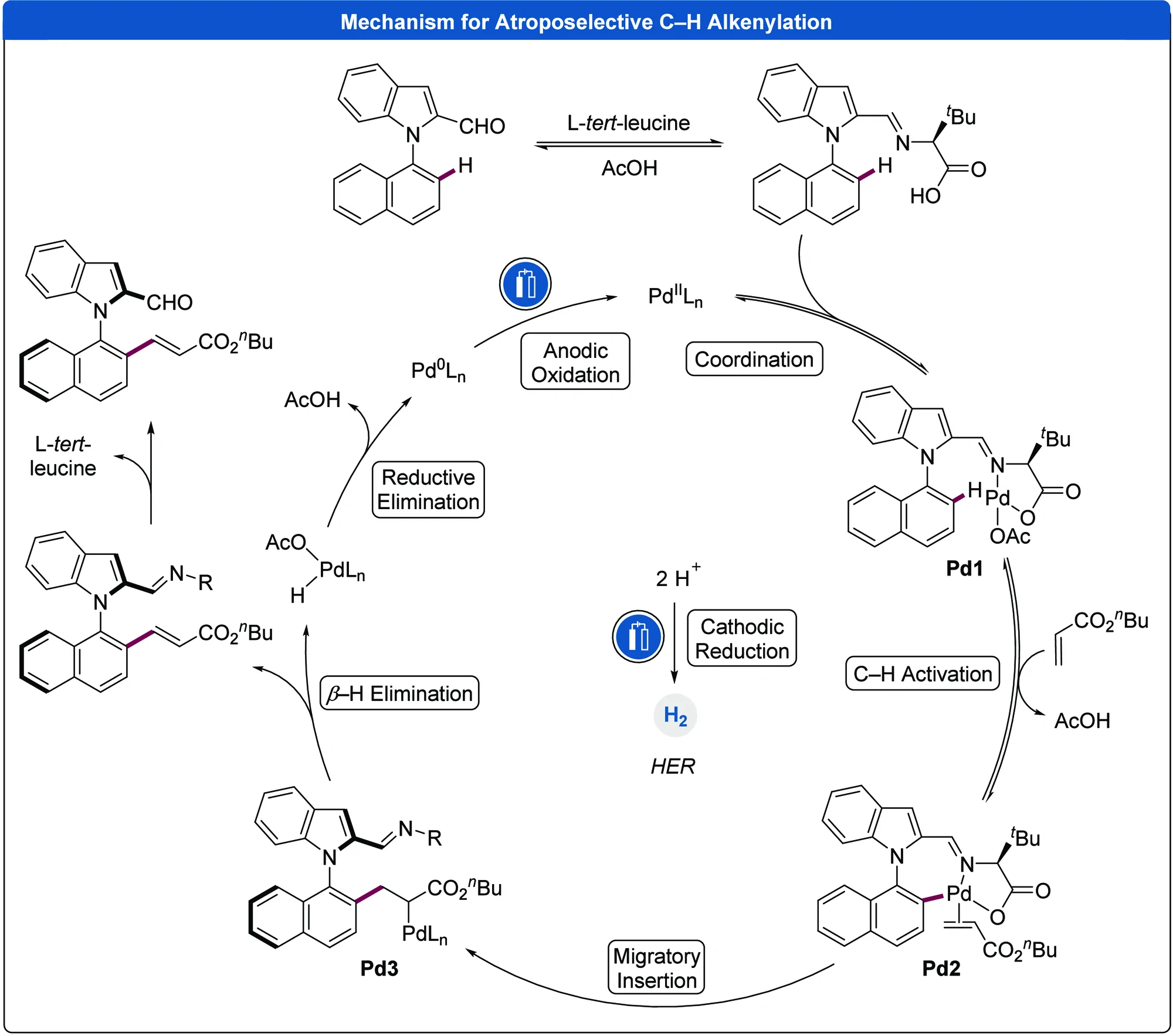
Proposed Mechanism for the Enantioselective Palladaelectro-Catalyzed C–H Alkenylation

Having established a robust and multifaceted strategy for atroposelective C–H activation *via* palladaelectro-catalysis, we focused on the asymmetric assembly of more challenging chiral C–N axes. An interesting motif is represented by acyclic anilides, which, in contrast to biaryls, possess an inherently higher degree of rotational freedom, making the enantioselective derivatization more challenging to control. Thus, exceedingly mild reaction conditions are essential, which was proven viable by our palladaelectro-catalyzed C–H activation strategy with (*S*)-5-oxoproline (**L1**) as chiral ligand (Scheme 5).^56^ Accordingly, the synthesis of a broad range of axially chiral anilides **11** was achieved with excellent chemical and optical yields (Scheme 5A). Detailed electroanalytical studies and *in-operando* spectroscopy provided insights into the *modus operandi*, indicating facile C–H activation and a dual role for benzoquinone: (i) it acts as a redox mediator in the oxidation of palladium, and (ii) it stabilizes palladium(0) intermediates through ligation. The resource-economic character of this transformation was demonstrated by efficient CCE using natural sunlight (Scheme 5B).

**Scheme 5:**
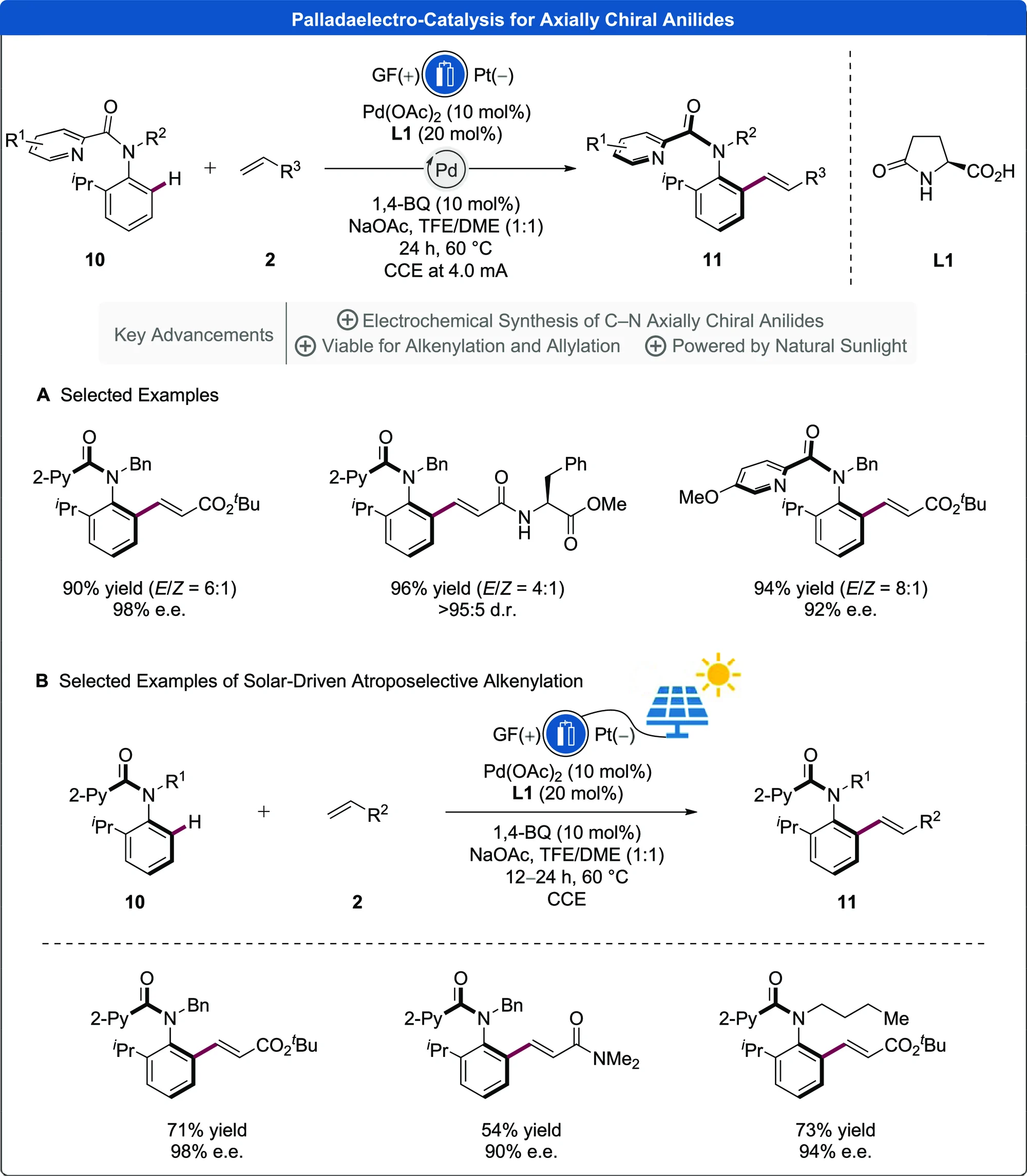
Palladaelectro-Catalyzed Atroposelective Construction of Chiral Anilides **11** ^a^

a Py = Pyridyl. BQ = Benzoquinone.

Despite significant advances in the development of enantioselective palladaelectro-catalyzed transformations, electrochemical transformations are inherently more complex due to an extended dimensionality of reaction parameters, influencing the overall efficacy and selectivity. An outstanding potential to elegantly streamline optimization campaigns in a resource-efficient manner can be attributed to emerging data-driven approaches, as represented by machine learning strategies.^57–61^ Moreover, merging predictive models with a closed-loop reaction design can significantly accelerate reaction optimization for method development.^62–64^ In collaboration with the Hong group, we were able to demonstrate the high value of such approaches in complex palladaelectro-catalyzed asymmetric transformations through the implementation of transition state knowledge.^58^ Beyond that, in 2024, we successfully disclosed an enantioselective palladaelectro-catalyzed annulation reaction through a ML-guided workflow (Scheme 6).^2^ The envisioned annulation of biaryl **1** with alkyne **12** initially furnished the annulated product **13** in a low yield of 9% and 98% enantiomeric excess with previously established reaction conditions.^1^ Considering key reaction parameters, such as electrode materials, electrolytes, solvents, and the applied currents or potentials, resulted in a total of 8640 possible reactions to be performed. The high-dimensional nature of this synthetic space was significantly reduced by the implementation of a ML-guided optimization workflow: testing a small, diverse set of reaction conditions and using the resulting yields to train a model that predicts outcomes across the full reaction space. Based on these predictions, new experiments were selected iteratively, first emphasizing diversity to explore broadly and then focusing on the most promising conditions, until optimal reaction conditions were identified with minimal experiments (Scheme 6A). Overall, the ML-guided strategy allowed us to identify optimal reaction conditions after performing solely 68 experiments in 12 rounds out of a total of 8640 possible combinations. The robustness of the obtained conditions was demonstrated by the enantioselective synthesis of versatile C–C and C–N axially chiral scaffolds **13** in excellent yields and enantioselectivities (Scheme 6B). Thus, an efficient enantioselective palladaelectro-catalyzed C–H activation was successfully optimized by ML guidance, whereby parameters, such as the electrode’s surface area, the conductivity of supporting electrolytes, and the solvent, had a substantial impact. As a return of the ML approach, electrolysis conditions were identified with most-user-friendly stainless steel anodes and cathodes, undermining that the extended chemical space in electrochemical settings can be grasped by data driven approaches.

**Scheme 6:**
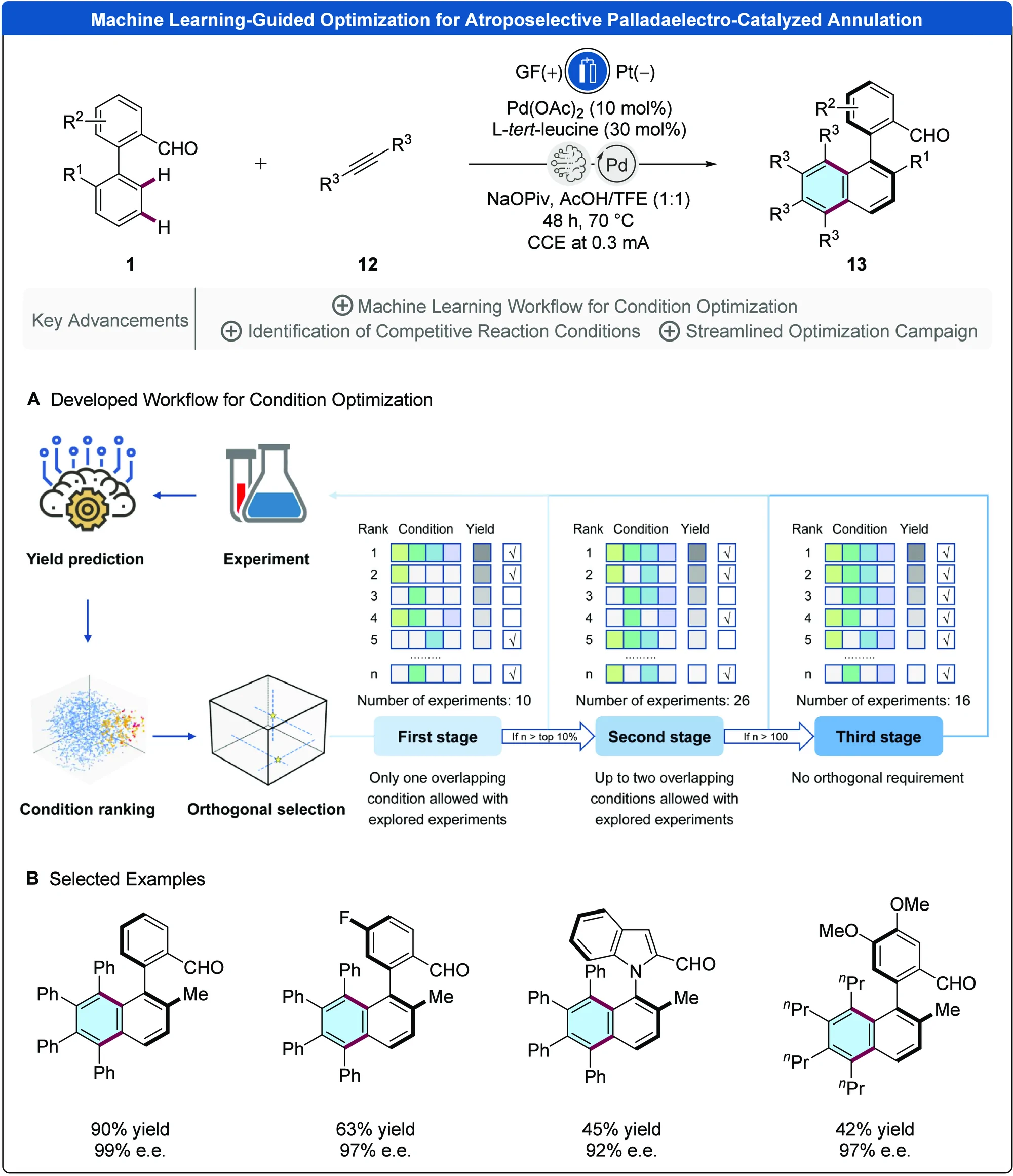
Machine Learning-Guided Optimization for Atroposelective Palladaelectro-Catalyzed C–H Annulation^a^

a Panel A taken from Hou et al.^2^ under the license CC BY-NC 4.0, without modifications.

Besides palladium, rhodium is a powerful transition metal, featuring notable reactivity along with outstanding chemo- and enantioselectivities in C–H activations.^65^ In this context, diverse chiral rhodium complexes with remarkable catalytic efficacies and selectivities surfaced with pioneering contributions by Murai^66^ and later Cramer,^67,68^ You,^69^ Antonchick and Waldmann^70^ as well as Perekalin.^71^ However, enantioselective and oxidative transformations relied thus far predominantly on chemical oxidants, typically stoichiometric silver­(I) salts.

Based on our previous experience in rhodaelectro-catalyzed oxidative C–H activations,^72–74^ we devised an enantioselective strategy for the rhodaelectro-catalyzed C–H spirocyclization, employing the chiral rhodium­(III)-catalyst **Rh1** (Scheme 7).^75^ The cyclization reaction between pyrazolone **14** and alkyne **12** proceeded efficiently, generating a diverse set of biorelevant spiropyrazolones **15** in good yields and enantioselectivities. Concurrently, Lu, You, and Mei reported a related elegant rhodaelectro-catalysis.^76^

**Scheme 7:**
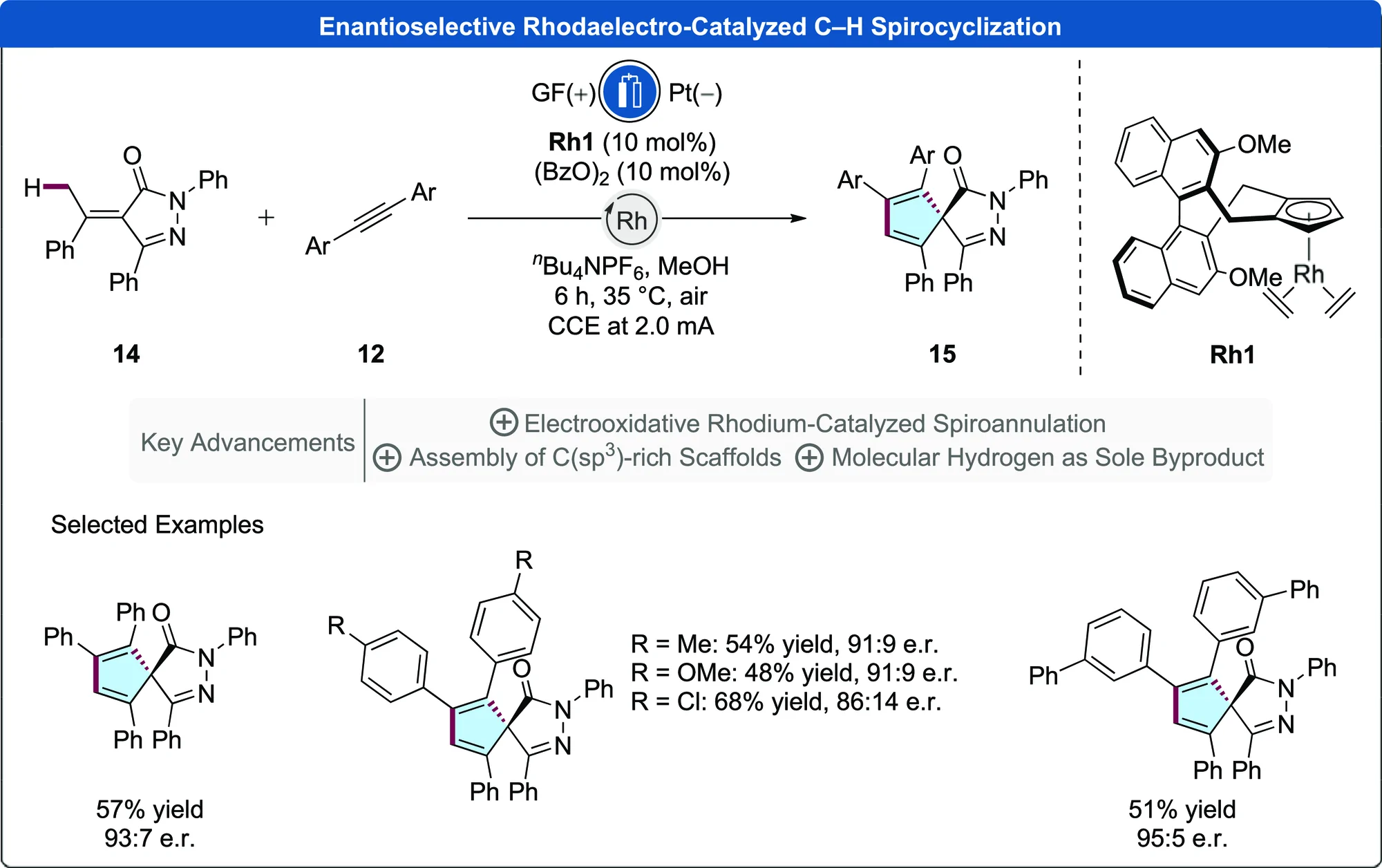
Rhodaelectro-Catalyzed C–H Activation for the Enantioselective Assembly of Spiropyrazolones **15**

Although chiral rhodium complexes demonstrated outstanding performances, their broader application is often limited by multistep syntheses of the chiral ligands, along with difficulties in accessing structural variations. To address this limitation, we considered chiral additives,^77^ such as chiral anions^78^ and carboxylic acids,^79,80^ or transient directing groups.^81^

Upon considerable experimentation, we achieved enantioselective rhodaelectro-catalysis using Cp*Rh­(OAc)_2_ with cinchonine (**C1**) as a naturally occurring, easily available source of chirality.^82^ Consequently, diverse chiral lactones **17** were constructed through an electrochemical domino catalysis platform, using simple benzoic acid derivatives **16** and acrylates **2** (Scheme 8). Our approach gave access to diverse chiral phthalides (Scheme 8A), being tolerant of different substitution patterns and functionalities. Detailed mechanistic studies allowed us to propose a reaction mechanism (Scheme 8B). First, carboxylate-assisted C–H activation with substrate **16** leads to the formation of cyclometalated Rh-complex **Rh3**. After coordination of alkene **2a**, migratory insertion and anodic oxidation enable the formation of seven-membered rhodacycle **Rh5**. Afterward, *β*-H elimination and reductive elimination furnish the alkenylated benzoic acid, followed by catalyst regeneration through anodic oxidation complemented by the HER. Finally, enantioselective lactonization through cinchonine-mediated oxa-Michael addition results in the desired product.

**Scheme 8:**
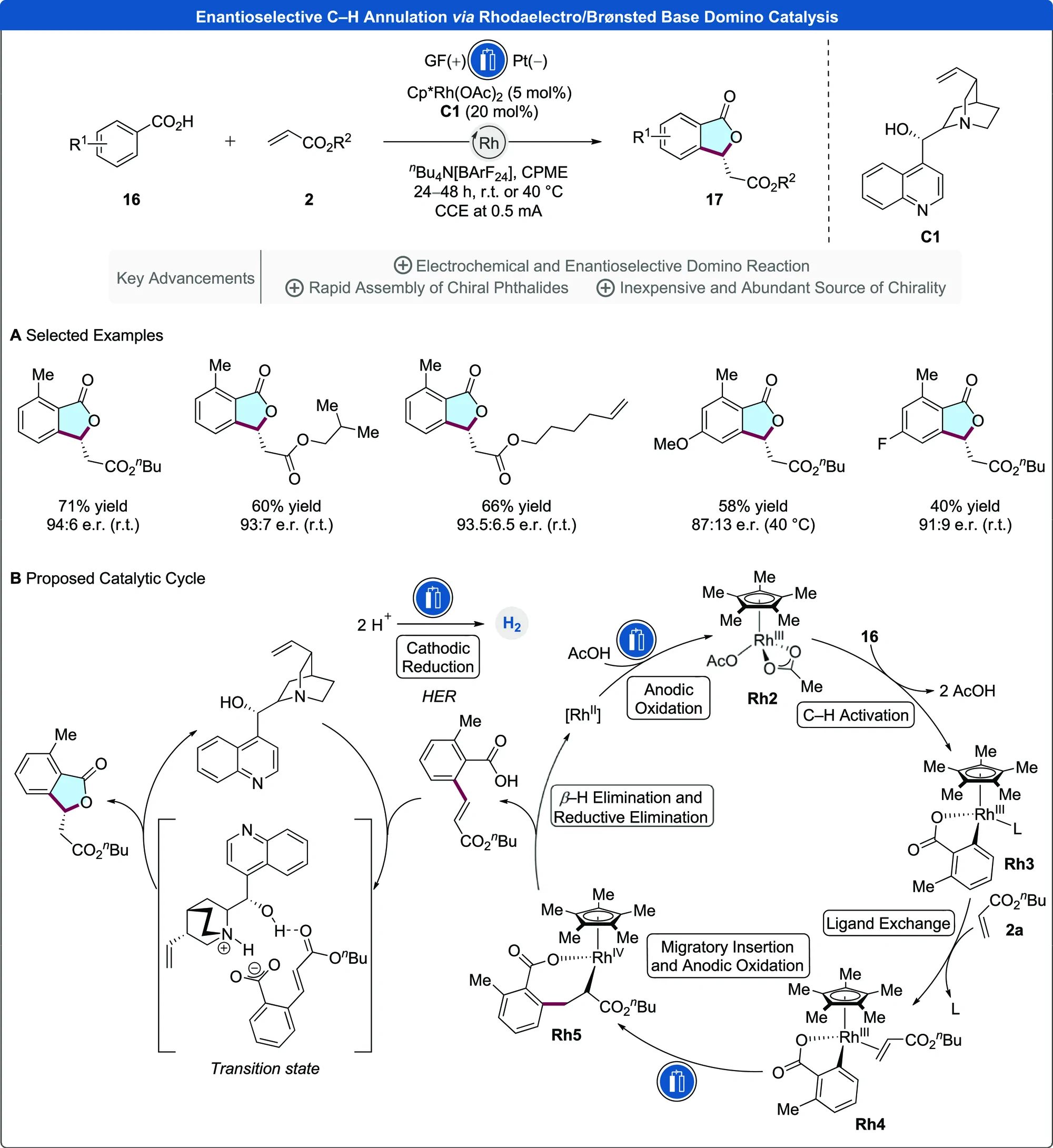
Enantioselective Construction of Chiral Lactones **17** Through Electrochemical, Rhodium/Chiral Brønsted Base Dual Domino Catalysis^a^

a CPME = Cyclopentyl methyl ether.

Given our more than two decades of experience in ruthenium catalysis,^83–87^ we were particularly intrigued to explore more cost-efficient and abundant ruthenium catalysts. Thus, we disclosed a versatile electrochemical approach for the construction of chiral molecular architectures (Scheme 9).^88^ The use of BenRu­(II) complexes^89,90^ allowed, on the one hand, for the assembly of axially chiral indoles **19** derived from anilines **18** through dehydrogenative annulation (Scheme 9A).^91^ On the other hand, the ruthenaelectro-catalysis manifold set the stage for the enantioselective spiroannulation with **14**, furnishing spirocycles **15** (Scheme 9B). Thereby, enantio-determining migratory insertion of alkyne **12** occurs after facile C–H activation, whereby attractive π–π interactions and repulsive steric interactions enable efficient enantioinduction. This is followed by reductive elimination and anodic reoxidation of the low-valent ruthenium intermediate.

**Scheme 9:**
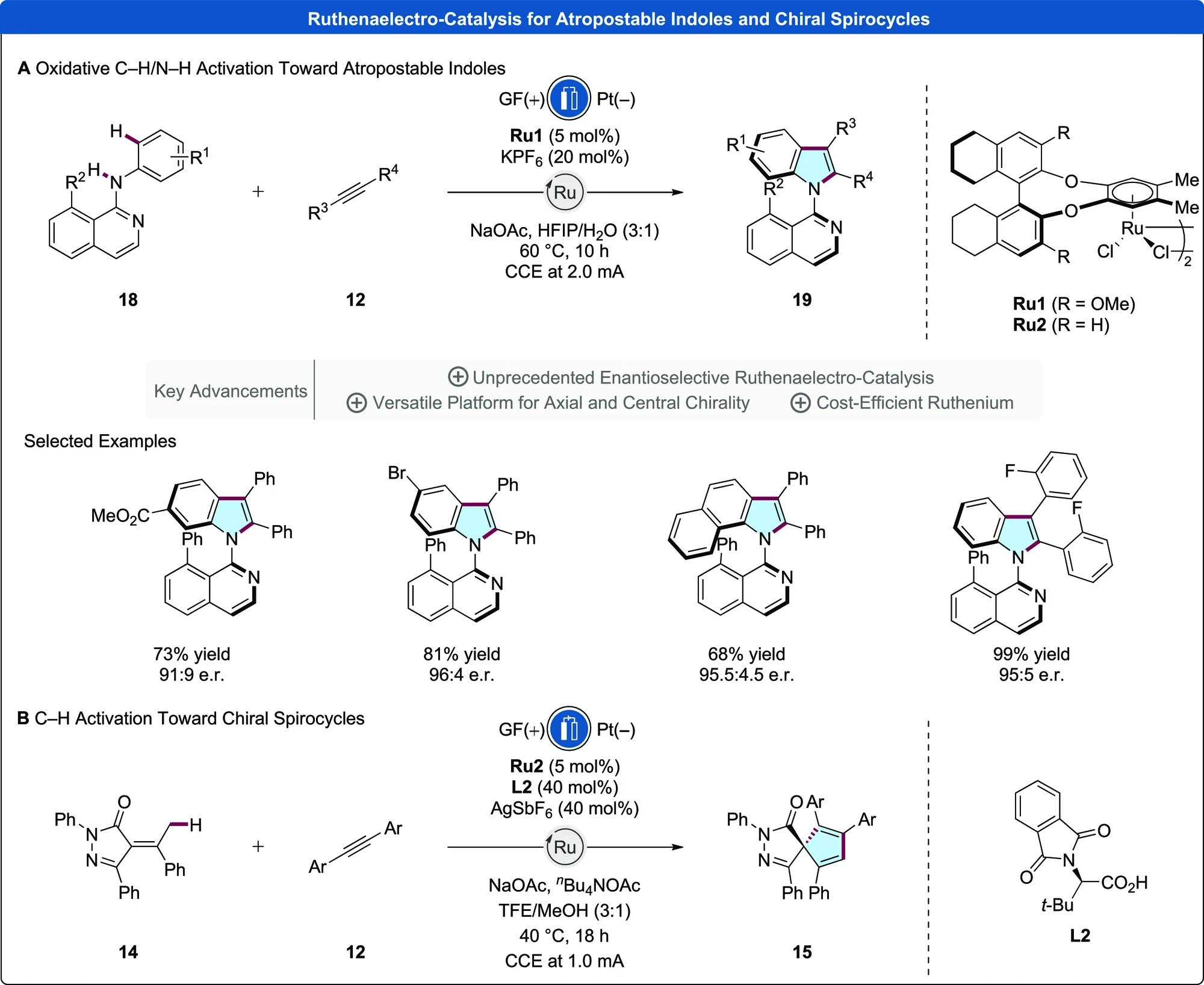
Ruthenaelectro-Catalyzed Platform for the Enantioselective Construction of Axially Chiral Indoles **19** and Spirocycles **15**

## 3 3d Metallaelectro-Catalysis

Catalysis is a fundamental pillar of sustainable molecular synthesis.^92^ Still, the choice of the transition metal represents a crucial factor in the evaluation of a process’s carbon footprint while simultaneously dictating its economic viability.^93^ Earth-abundant metal catalysts prevail over their noble congeners with broad availabilities, typically lower toxicities, and cost-effectiveness.^30,92,93^ Due to the pronounced redox flexibility, the use of 3d transition metals in electrocatalysis enables the exploration of their full potential with a synergistic flux of protons and electrons *via* SET processes. Interdisciplinary efforts in recent years enabled selective electrochemical manipulations, demystifying its applicability and transforming 3d metallaelectro-catalysis into a versatile toolbox for resource-economic synthesis.^30,31^ Nevertheless, the more labile metal–ligand bonds and the high reactivity complicate the use in enantioselective metallaelectro-catalysis, rendering efficient stereoinduction under electrooxidative conditions a daunting task with exceptional potential.

Cobaltaelectro-catalysis is positioned at the forefront in electrocatalyzed C–H activation, since early reports by our group on C–H oxygenation, nitrogenations, and annulations,^31,94,95^ resulting in a plethora of diverse transformations (Scheme 10). Elaborate mechanistic investigations shed light on the key cobaltacycle intermediate formed through anodic oxidation, which was independently prepared, unraveling the dual role of electricity as the terminal oxidant and for enabling a cobalt­(III/IV)-oxidation-induced reductive elimination.^36^ A crucial feature of the cobaltacycle is a second substrate being coordinated in a monoanionic, bidentate fashion. Simultaneously, the scientific community, spearheaded by Ackermann^96^ and later Cramer,^97^ Matsunaga,^98^ and Shi,^99^ devised high-valent cobalt­(III) catalysis as a robust tool in enantioselective catalysis. Thus, hemilabile monoanionic chiral ligand motifs were identified, namely, chiral carboxylic acids, amino acids, salox ligands, and phosphoric acids (Scheme 10). Thus, we became interested in exploring the power of enantioselective cobaltaelectro-catalysis.

**Scheme 10:**
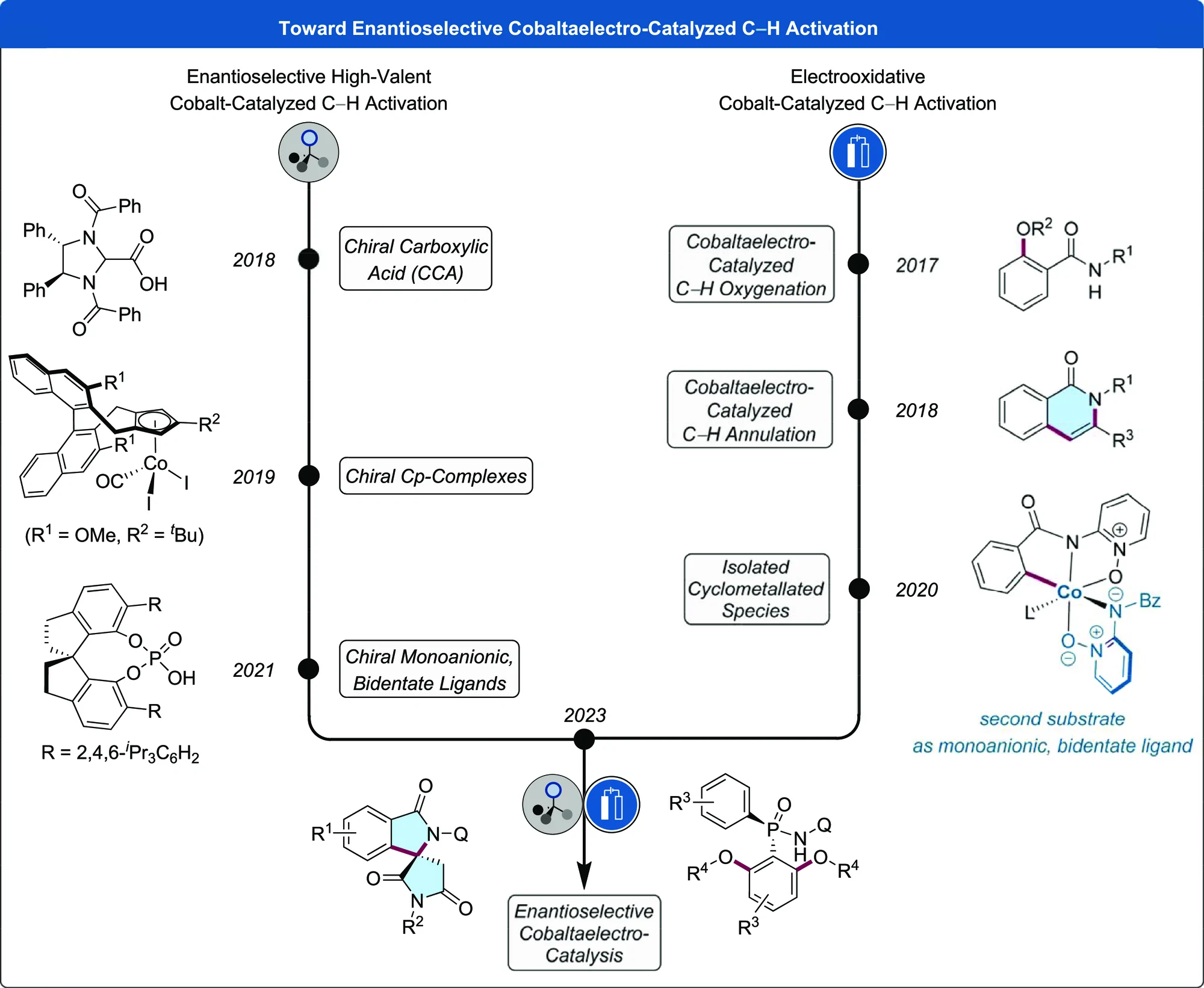
Progress in Enantioselective Cobaltaelectro-Catalyzed C–H Activation

As a result, we successfully realized the merger of cobaltaelectro-catalyzed C–H activation and enantioselective catalysis with an array of distinct transformations, being coupled to the HER with notably high Faradaic efficiency under tolerance of air and moisture.^3^ Thereby, the construction of all-carbon quaternary stereogenic centers was orchestrated *via* spirocyclization, affording scaffolds **22** with remarkable chemo-, position-, and enantioselectivities (Scheme 11). In stark contrast to previous reports on chemical oxidation,^100^ the electrooxidative strategy used commercially available chiral phosphoric acid (CPA) **L3** deprived of the 3,3′-substitution pattern.^3^ A broad range of sensitive functional groups and practical synthesis handles were fully tolerated by the developed conditions (Scheme 11A). Mechanistic investigation corroborated the hypothesis in terms of the cyclometalated cobalt­(III) species **Co1**, obtained *via* anodic SET oxidation and displaying an additional X,L-type ligand in a bidentate fashion (Scheme 11B). Upon anodic oxidation, product **22** was obtained within a stoichiometric transformation. An enantioinduction with a similar level of selectivity as compared to the catalysis manifold by the action of the chiral phosphoric acid **L3** was observed. Thereby, oxidation-induced spirocyclization after migratory insertion was supported by detailed mechanistic studies, including *in-operando* high-resolution electrospray-mass spectrometry (HRMS) and spectroelectrochemical investigations.

**Scheme 11:**
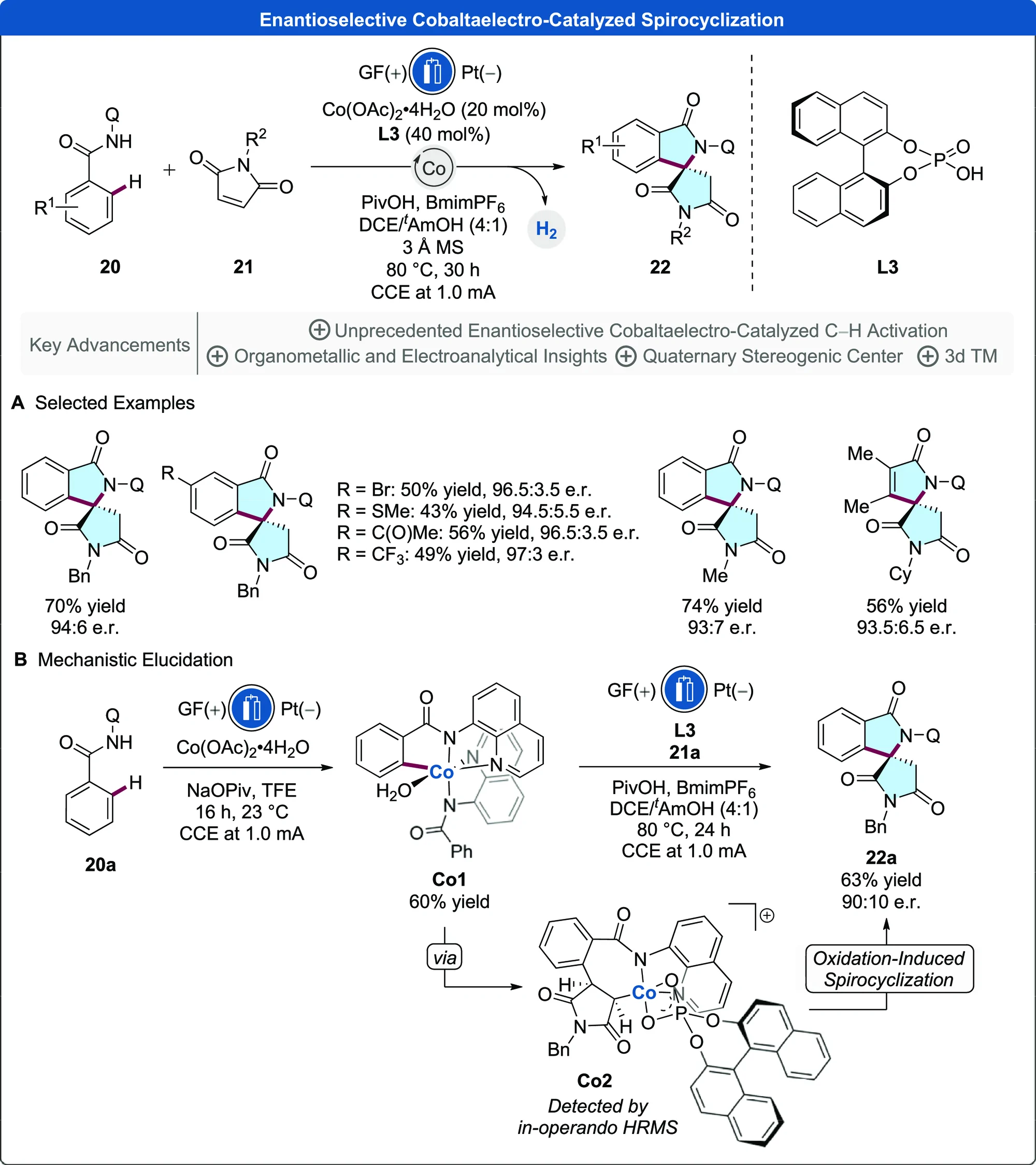
Cobaltaelectro-Catalyzed Spirocyclization for the Construction of Quaternary All-Carbon Centers in Scaffolds **22** ^a^

a Q = 8-Quinolinyl. Bmim = 1-*n*-Butyl-3-methylimidazolium.

These insights were subsequently harnessed for a vast extension of viable conversions (Scheme 12), benefiting from the Bolm-salicycloxazoline (Salox) ligand motif.^3,101,102^ Alkynoates **23** were thus transformed through a cascade reaction, involving dehydrogenative C–H annulation, into atropostable isoquinolones **24** (Scheme 12, path a). Hence, the strategy meets the continuously rising interest in axial chirality by, among others, the pharmaceutical industry.^53,54^ Further, the simultaneous construction of two adjacent stereogenic centers in a single manipulation proved viable with unactivated alkenes **5** (Scheme 12, path b), exhibiting excellent diastereoselectivity control toward chiral isoquinolones **25**. The electrocatalysis robustness was further mirrored by the desymmetrization of phosphinamides *en route* to phosphorus-centered point chirality, extending the accessible chemical space. Thus, cobaltaelectro-catalyzed C–H oxygenation was made possible in an enantioselective fashion for crafting chiral phosphinamides **27** (Scheme 12, path c). Additionally, dehydrogenative C–H annulation of a set of alkynes **12** and **21** provided efficient access to fused polycyclic *P*-stereogenic compounds **28** and **29** under simultaneous C–C, C–N, and C–O bond formation. Noteworthily, the cobaltaelectro-catalysis’ efficiency was maintained using solar energy^11,103^ as the sole power supply, empowering the vast potential toward sustainable molecular synthesis.^3^

**Scheme 12:**
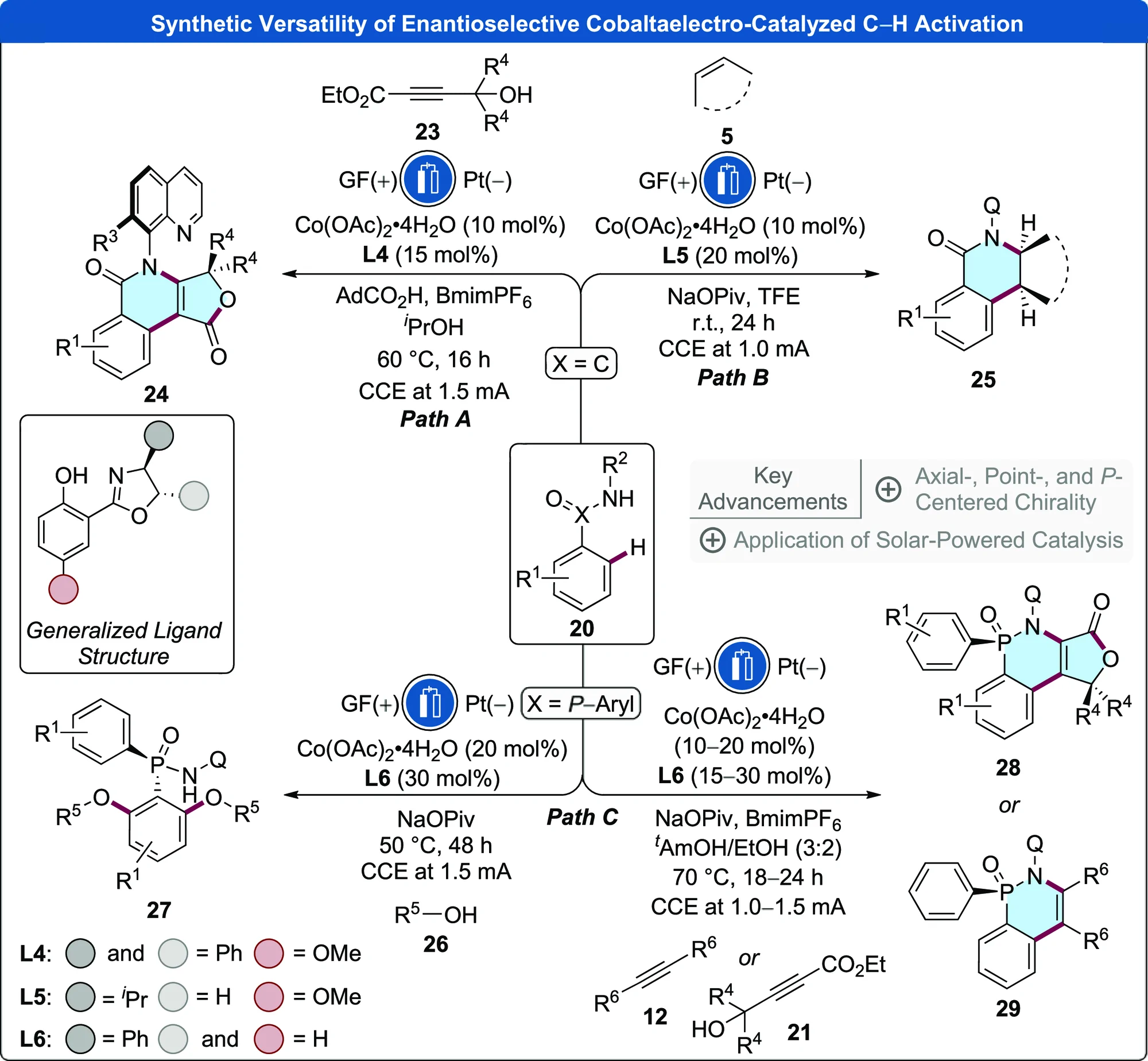
Enantioselective Cobaltaelectro-Catalyzed C–H Activation for the Preparation of Axial-, *C*-Point-, and *P*-Based Chirality

With ameliorated mass and heat transfer, precisely controllable reaction parameters, and comparably short interelectrode distances, continuous flow operations set the stage for, among others, fast kinetics, improved selectivities, diminished risk for overoxidation, and reduced amounts of supporting electrolytes.^17,104–106^ Coherent progression from academic discovery to scaled processing is viable, obviating the prerequisite for laborious reoptimizations.^107–109^ While direct scalability was already proven in batch, subsequent investigations further showed that the cobaltaelectro-catalysis proved to be viable in continuous flow (Scheme 13).^110^ Thereby, allenes **30** were investigated as unique coupling partners, giving rise to the atroposelective preparation of isoquinolones **31** on decagram scale.

**Scheme 13:**
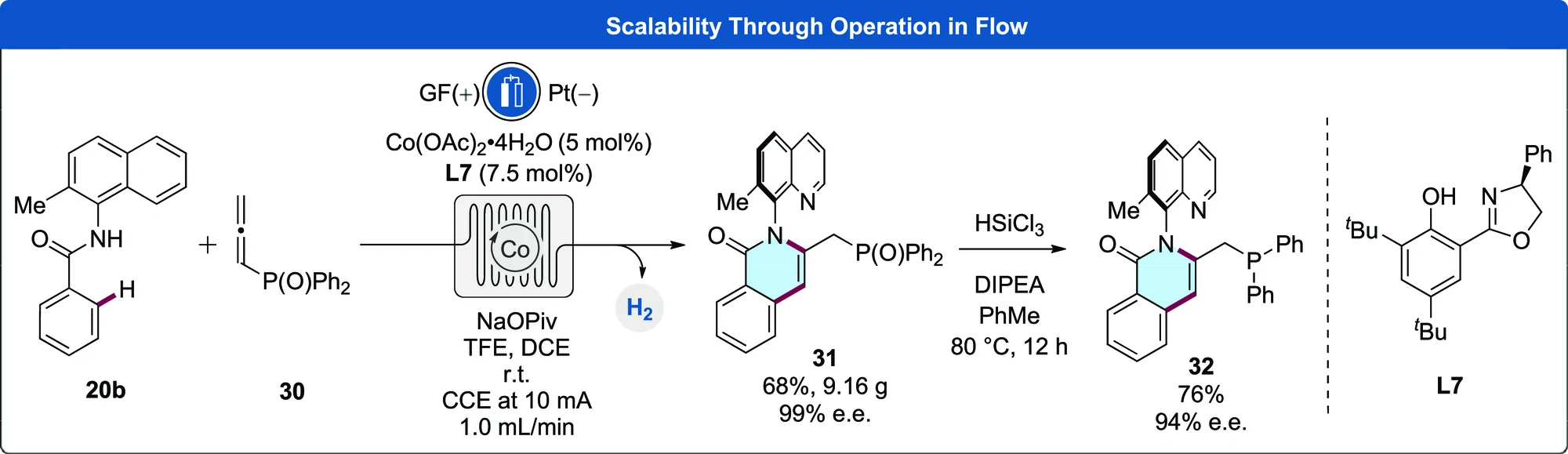
Decagram-Scale Enantioselective Cobaltaelectro-Catalyzed C–H Annulation in Flow for the Preparation of Atropostable Isoquinolones **31**

Subsequently, the transition toward higher degrees of structural complexity was achieved by the simultaneous construction of central and axial chirality in a single step with excellent enantio- and diastereo-discrimination (Scheme 14).^111^ Diverse cyclic and acyclic olefins **5** were amenable for the construction of scaffolds **25**, giving access to two stereogenic centers and a chiral axis through dehydrogenative C–H/N–H annulation (Scheme 14A). Furthermore, the formation of molecular hydrogen through cathodic proton reduction was proven by qualitative and quantitative monitoring of the gas evolution during the electrolysis. The Faradaic efficiency for the HER was >80%, significantly higher than typical quantum yields of photoredox-catalyzed transformations. Based on our continuous mechanistic studies,^36,112,113^ a catalytic cycle was proposed (Scheme 14B) to commence with a facile C–H activation *via* a base-assisted pathway mediated by cobalt­(III). The resulting intermediate **Co4** was independently prepared and fully characterized. Then, an enantio-determining migratory insertion and diastereo-determining reductive elimination lead to formation of the desired product **25**. Subsequently, the concomitantly formed cobalt­(I) species **Co6** is anodically oxidized, ensuring catalytic turnover. Enantioinduction is primarily achieved through attractive π–π interactions between substrate and ligand. Other cobaltaelectro-catalyzed annulation reactions with alkenes or alternative π-system-based coupling partners follow a strongly related mechanistic pathway, while C–heteroatom bond formations rely on an oxidation-induced reductive elimination *via* cobalt­(IV).^36^ These findings on enantioselective cobaltaelectro-catalyzed C–H activations have thereafter inspired elegant studies by Shi,^99,114,115^ Niu,^116–120^ Ling,^121,122^ and Zeng^123^ for exciting expansions, thus mirroring its versatility.

**Scheme 14:**
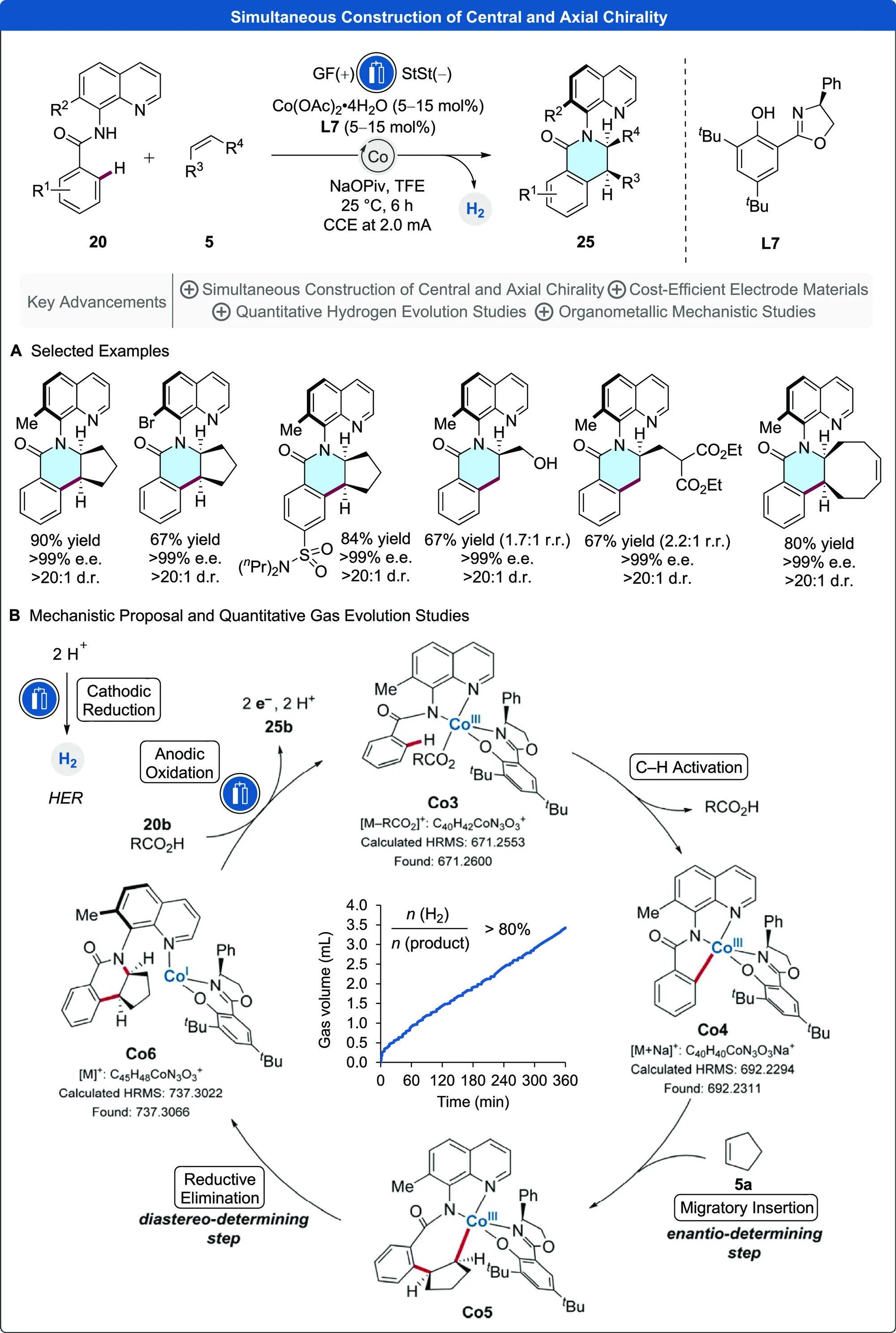
Selective Assembly of Point and Axial Chirality *via* Cobaltaelectro-Catalyzed C–H Annulation^a^

a Figure concerning gas evolution studies taken from von Münchow et al.^111^ under the license CC BY-NC 4.0, without modifications.

Paramount advances in catalysis research frequently stem from *de novo* preparations and modifications of chiral ligands, providing a resilient driving force toward innovations in enantioselective metallaelectro-catalysis.^124,125^ Thus, our group devised a program on the development of distinct κ^2^-*N*,*O*-preligands in conjunction with in-depth insights into structure–property relationships *via* data-driven parametrization and prediction to precisely maneuver sophisticated reactivity (Scheme 15).^126,127^ Thereby, novel enol, amide, urea, and thiourea ligands composed of an oxazoline backbone were developed, which are easily accessible in a highly modular fashion. First, enol-derived preligand **L8** was exploited for the construction of indanes **34**, consisting of three consecutive stereogenic centers and an azo moiety (Scheme 15A).^126^ This reactivity was viable by an intramolecular addition/elimination on the electrophilic hydrazone directing group, thus setting the stage for a scarce C­(sp^3^)–C­(sp^3^) bond formation in an enantio- and diastereoselective manner. A related conversion was likewise viable.^128^ Importantly, a comprehensive understanding of the effect of various tested ligands’ structural, vibrational, and electronic modularity was ensured *via* theoretical parametrization with DFT-based descriptors, establishing a structure-enantioselectivity relationship for future applications and advancements.^134^ Thereby, distinct natural bonding orbital (NBO) charges on the ligand and interaction energies (IEs) between ligand and substrate were found to be decisive. Further notable features include an efficient scale-up, reactivity driven by natural sunlight, and a successful reduction of the azo functionality, providing access to chiral amines.

**Scheme 15:**
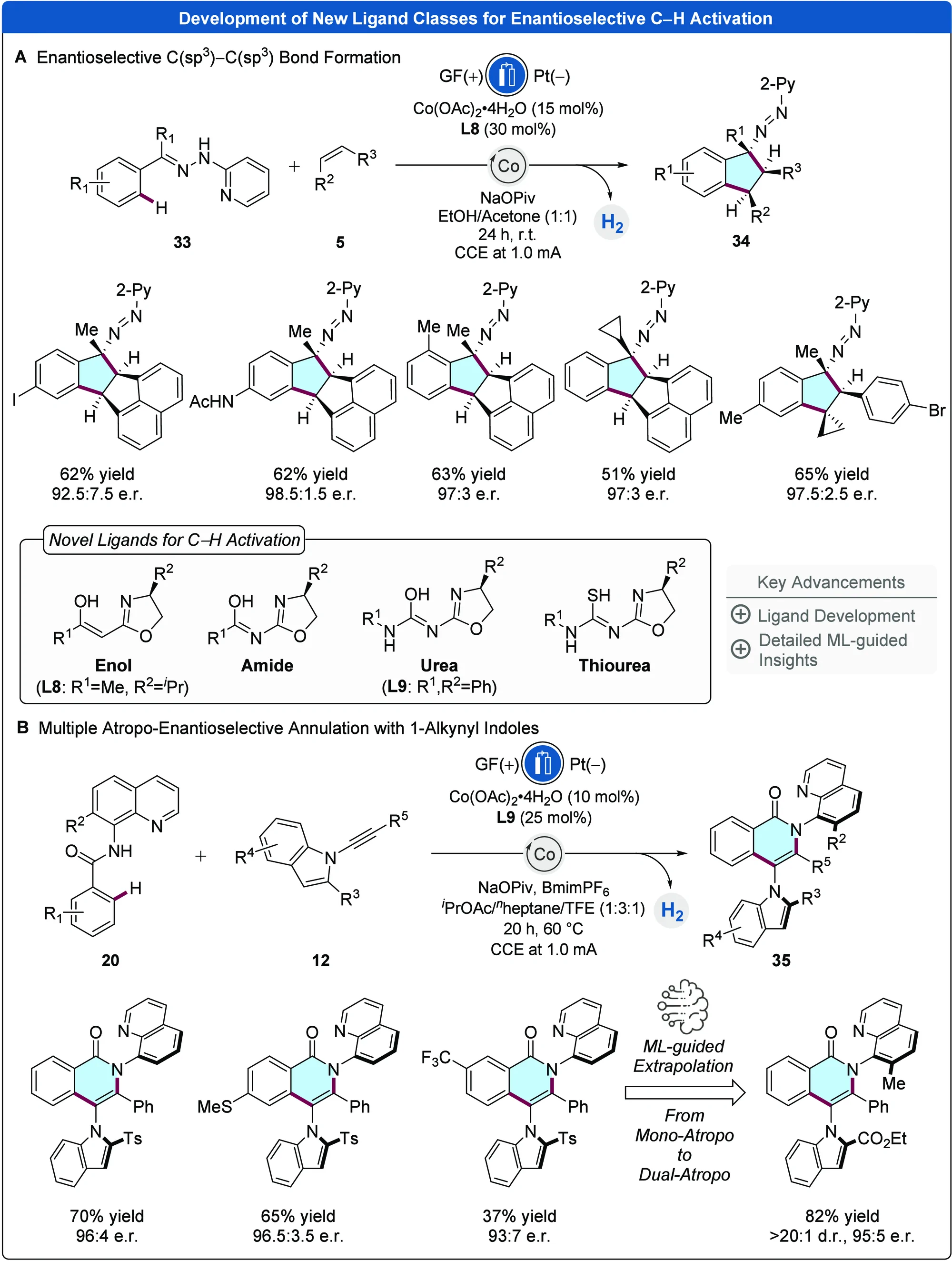
ML-Guided Ligand Development for the Selective Assembly of Point and Axial Chirality *via* Cobaltaelectro-Catalyzed C–H Annulation

Subsequently, a challenging C–H/N–H annulation with 1-alkynyl indoles **12** was achieved, electrochemically crafting axial chirality by exploiting novel urea- and thiourea-based preligands (Scheme 15B).^127^ Crucially, a multivariate linear regression (MVLR) model was developed, disclosing decisive features (dihedral angle, NBO charges, and sterimol parameters from the ligand) that influenced catalytic efficacy and atroposelectivity; hence, setting the stage for future developments. Further, this approach featured predictive power, improving enantioselectivity and opening the pathway to an unprecedented construction of two different chiral C–N axes.

Besides cobalt, alternative 3d transition metals have been explored in electrochemical C–H activation, ameliorating the environmental implications of established approaches.^30^ The recent surge in sustainable 3d transition metal-catalyzed C–H activation has promoted the use of earth-abundant copper as an efficient catalyst in electrochemical settings.^129,130^ On this premise, an enantioselective approach proved indeed viable for the preparation of chiral ferrocene derivatives through cupraelectro-catalyzed C–H activation (Scheme 16).^131^ Thereby, commercially available BINOL (**L10**)^132–134^ was leveraged in C–H phosphorylations and sulfonylations, selectively constructing the planar chiral scaffolds **38** and **40**. The exceedingly mild conditions allowed for broad tolerance of sensitive functional groups, underlining the robust nature of metallaelectro-catalysis. Mechanistically, the transformations were proposed to proceed through base-assisted C–H activation with 1,1,3,3-tetramethylguanidine (TMG), followed by coordination of substrates **40** or **42** and reductive elimination. Turnover is warranted by anodic oxidation of the reduced copper intermediate.

**Scheme 16:**
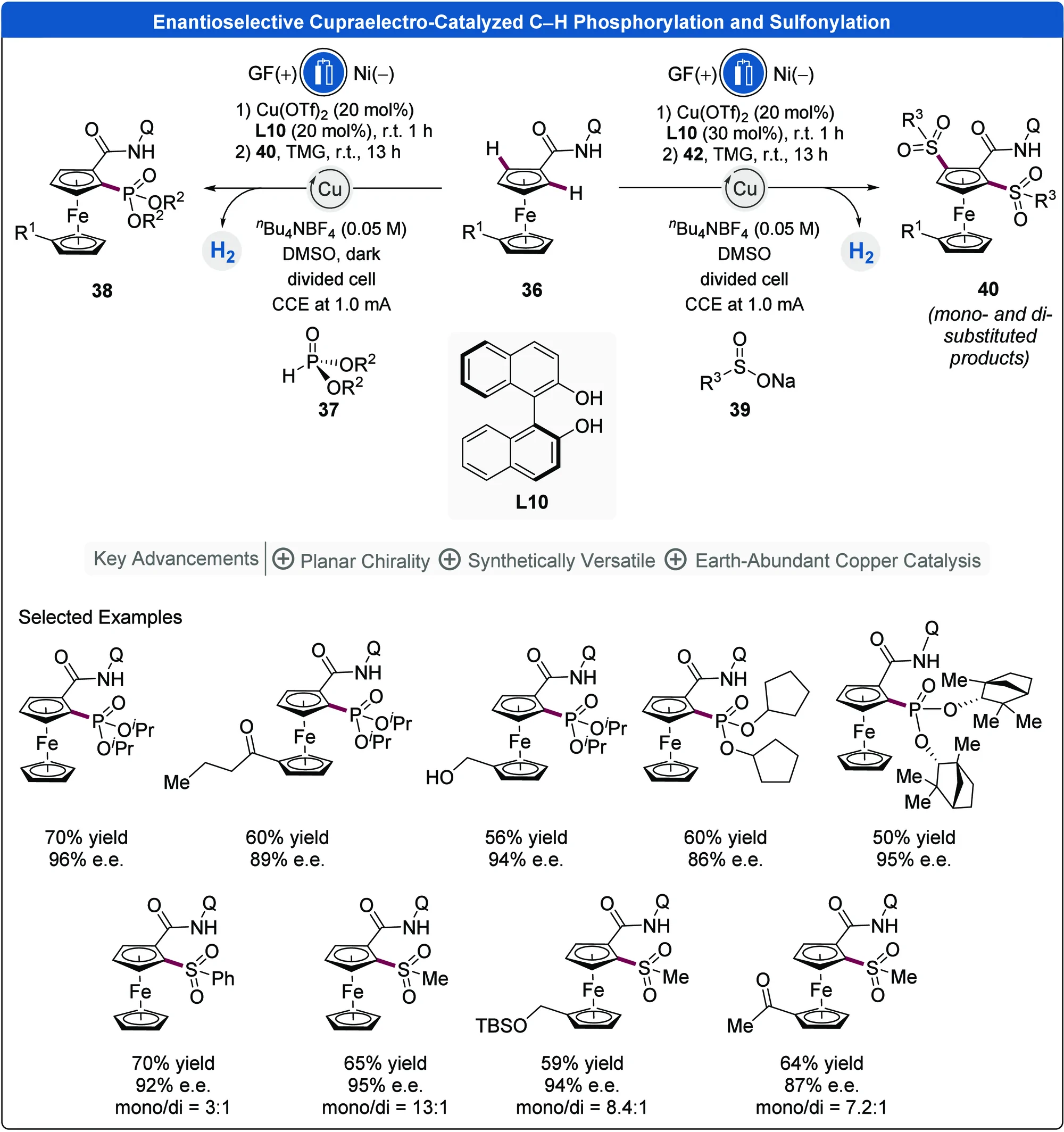
Cupraelectro-Catalyzed Enantioselective C–H Activation Toward Planar Chirality in Ferrocene Deriviatives **38** and **40** *via* C–H Phosphorylation and Sulfonylation

Nickel catalysis is of topical interest due to its natural abundance, low toxicity, and complementary reaction profiles.^135–137^ Advances in enantioselective catalysis were severely limited to its low-valent regime, requiring sensitive precatalysts, a meticulously controlled inert reaction environment, and pyrophoric organoaluminum reagents for a beneficial heterobimetallic regime.^138–141^ The latter hurdle was addressed in 2019 with an aluminum-free nickel-catalyzed hydroarylation.^142^ Still, the exploration of nickel’s redox flexibility in an enantioselective electro-setting remained unexplored.

In stark contrast, our group devised high-valent nickelaelectro-catalyzed approaches for C–H alkoxylation,^37^ amination,^143^ and phosphorylation,^144^ as well as a multiple dehydrogenative coupling approach for pyrrole synthesis,^145^ using air-stable precatalysts in the absence of stoichiometric chemical oxidants. The streamlined access to high-valent nickel intermediates by electrochemical means represented a promising development to broaden the applicability of enantioselective nickel catalysis. The breakthrough was achieved in 2025 with an unprecedented report on the efficient desymmetrization of strained bicyclic olefins **41** toward C­(sp^3^)-rich scaffolds **42** *via* enantioselective nickelaelectro-catalyzed C–H activation (Scheme 17), crafting several contiguous stereogenic centers in a single step with excellent control of the diastereo- and enantioselectivity.^4^

**Scheme 17:**
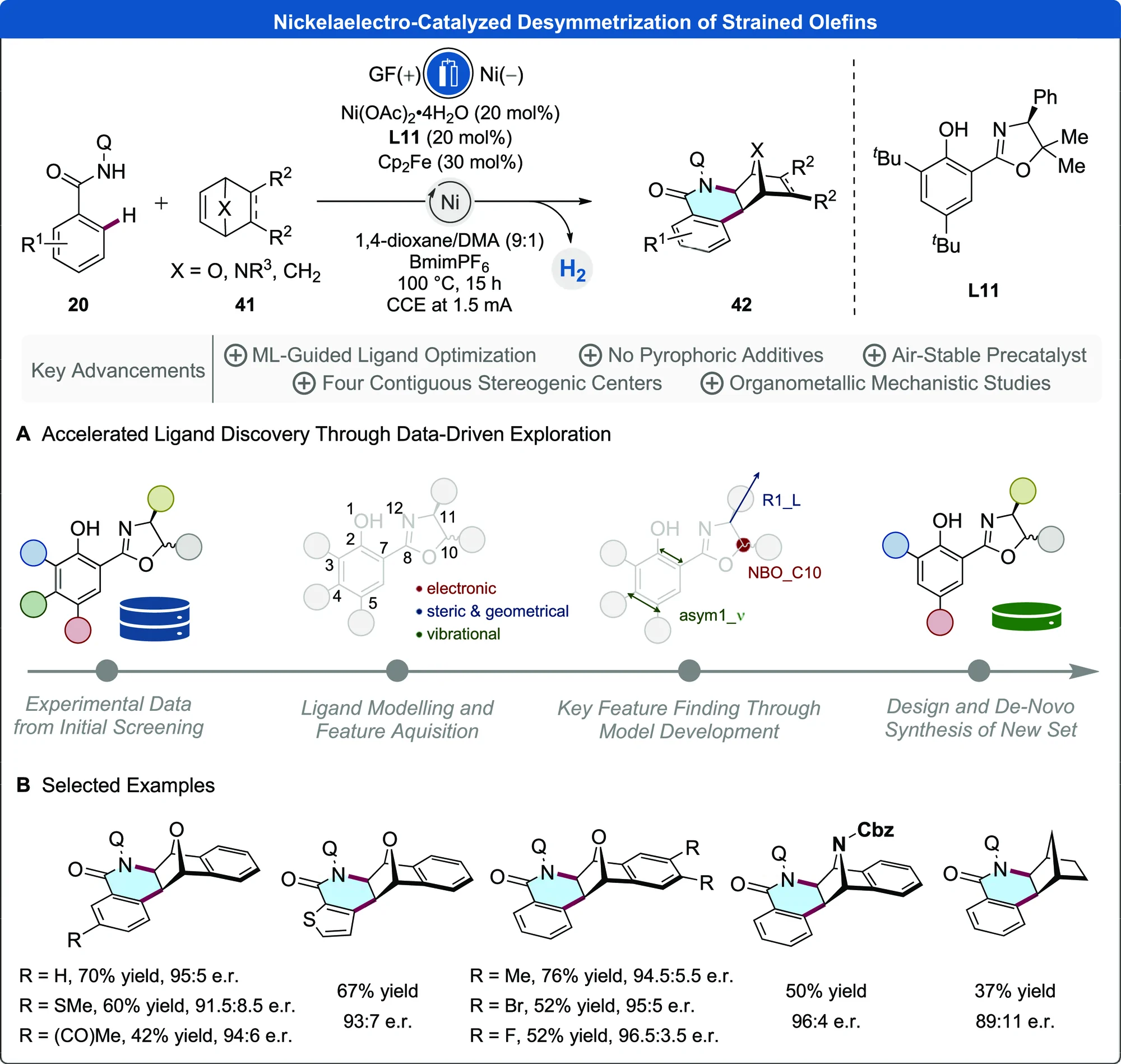
Nickelaelectro-Catalyzed Enantioselective C–H Activation for the Desymmetrization of Olefins **41** *via* Dehydrogenative Annulation

Enantioinduction was achieved on a moderate level in initial empirical studies. Therefore, a data science approach was chosen for ligand optimization, reflecting the synergistic dynamic of interdisciplinary efforts toward accelerated discovery (Scheme 17A). DFT-derived descriptors were explored to identify decisive ligand features *en route* to efficient selectivity control. By leveraging a tree-based regression model on the screening data for the feature importance analysis, three vital features were unraveled, involving the sterimol parameter of C11 (R1_L), the asymmetric stretching mode 1 of the benzene moiety, and the NBO charge at C10 (NBO_C10). Later, the best selected gradient boosting regression (GBR) model confirmed the importance of the same set of features. Based on this crucial insight, novel ligand designs were proposed and experimentally studied, resulting in **L11** as the best candidate, which provided efficient control in this sophisticated conversion through noncovalent π–π and CH−π interactions. Accordingly, a broad wealth of differently substituted motifs was efficiently converted, including aza- and carbon-bridged bicycles with reduced selectivity (Scheme 17B). A very similar transformation was subsequently reported.^146^

Subsequently, extensive organometallic and electroanalytical studies shed light on the *modus operandi* (Scheme 18), setting the stage for future advancements.^4^ Deuterium labeling experiments highlighted the ability of nickel­(II) to participate in reversible C–H activation. Still, the isolated cyclometalated complex **Ni1** proved ineffective for stoichiometric product formation, suggesting the necessity for oxidation toward product formation (Scheme 18A, centered pathway). Since ferrocene exhibited an advantageous effect on the reaction efficacy, it was proposed to act as a redox mediator, oxidizing nickel in a homogeneous fashion after heterogeneous anodic oxidation. Therefore, **Ni1** was exposed to ferrocenium hexafluorophosphate as a homogeneous oxidant, enabling stoichiometric product release and verifying both hypotheses (Scheme 18A, upper pathway). Conducting this reaction without the coupling partner led to the formation of the nickel­(III) species **Ni2**, which was isolated and characterized (Scheme 18A, lower pathway). In the absence of oxidative conditions, **Ni2** released the product **42a** stoichiometrically, indicating feasible reductive elimination from nickel­(III) and ruling out an oxidation-induced reductive elimination, as observed for previous nickelaelectro-catalyzed transformations.^37,137^ The spectroelectrochemical (SEC) investigation of the chronoamperometric oxidation of **Ni1** showcased a mirror-image behavior of **Ni2**’s reduction, suggesting facile interconversion of both potential intermediates by SET. The findings were summarized in a mechanistic proposal (Scheme 18B). Thereby, facile C–H activation leads to the cyclometalated species **Ni4**, followed by SET oxidation toward the mimicked intermediate **Ni2**. Then, migratory insertion of the bicyclic olefin **41a** leads through intermediate **Ni5** and subsequent reductive elimination to the experimentally observed product.

**Scheme 18:**
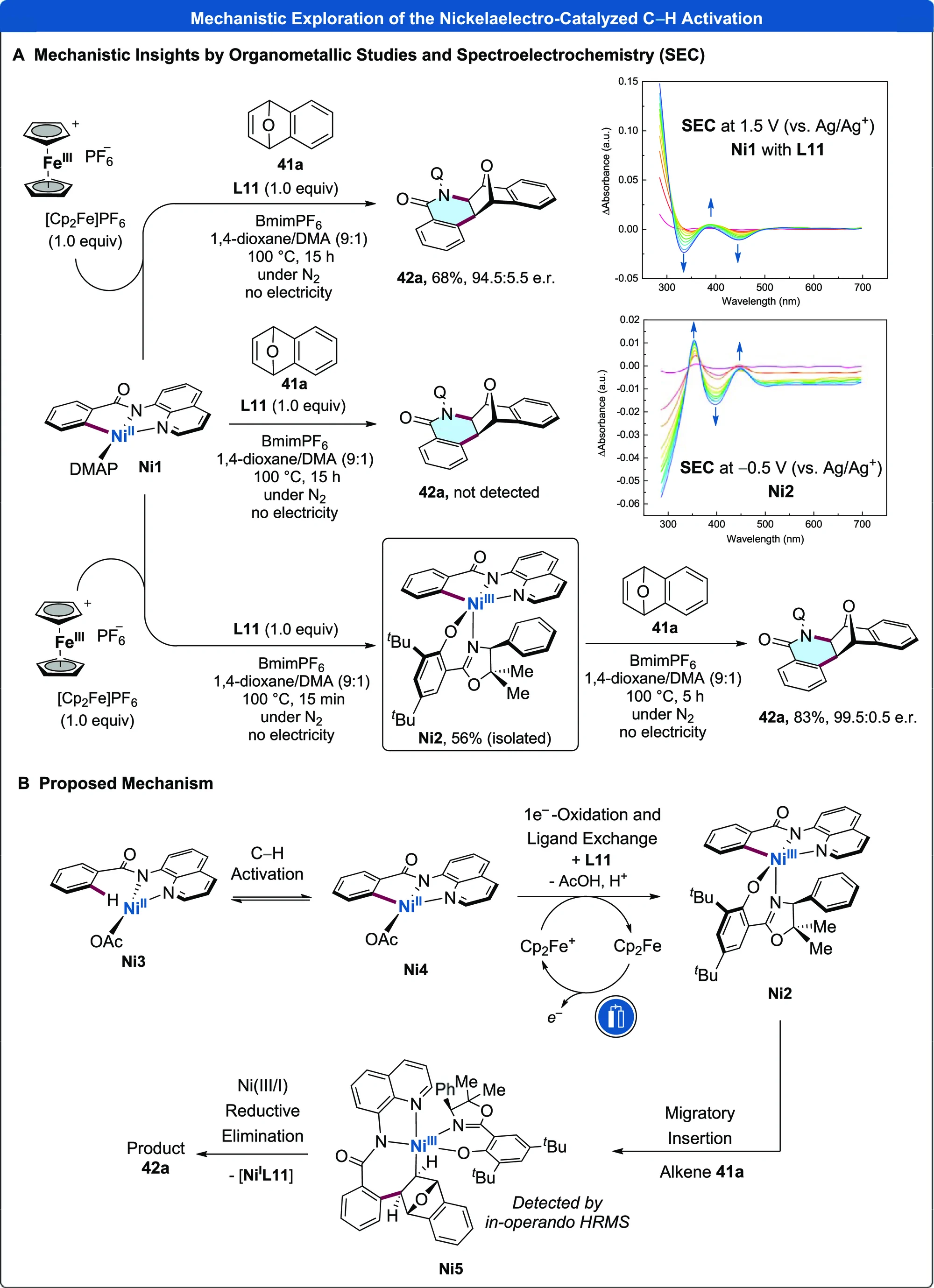
Deep Mechanistic Insights into the Nickelaelectro-Catalyzed Desymmetrization^a^

a SEC figures taken from von Münchow et al.^4^ under the license CC BY-NC 4.0, with modified description.

Interestingly, besides the control of the diastereo- and enantioselectivity, an altered chemoselectivity was observed by switching the precatalyst to cobalt, undermining the complementary reaction profiles (Scheme 19).^4^ Thus, the carboacylation product **43** was afforded. Organometallic studies indicated the initial formation of a related intermediate, as in the nickel-catalyzed variant, after C–H activation and migratory insertion. The chemodivergent reaction pathway was subsequently unraveled by computational investigations (Scheme 19A). Thereby, it was found that the cobalt-consisting intermediate exhibits an increased nucleophilicity at the cyclometalated carbon due to elevated electropositivity compared to nickel. Hence, the intramolecular nucleophilic attack at the amide moiety occurs in contrast to the previously observed reductive elimination. The chemodivergent behavior was then broadly tested with differently decorated benzamides and olefins, tolerating various sensitive functional groups in high enantio- and diastereoselectivities (Scheme 19B).

**Scheme 19:**
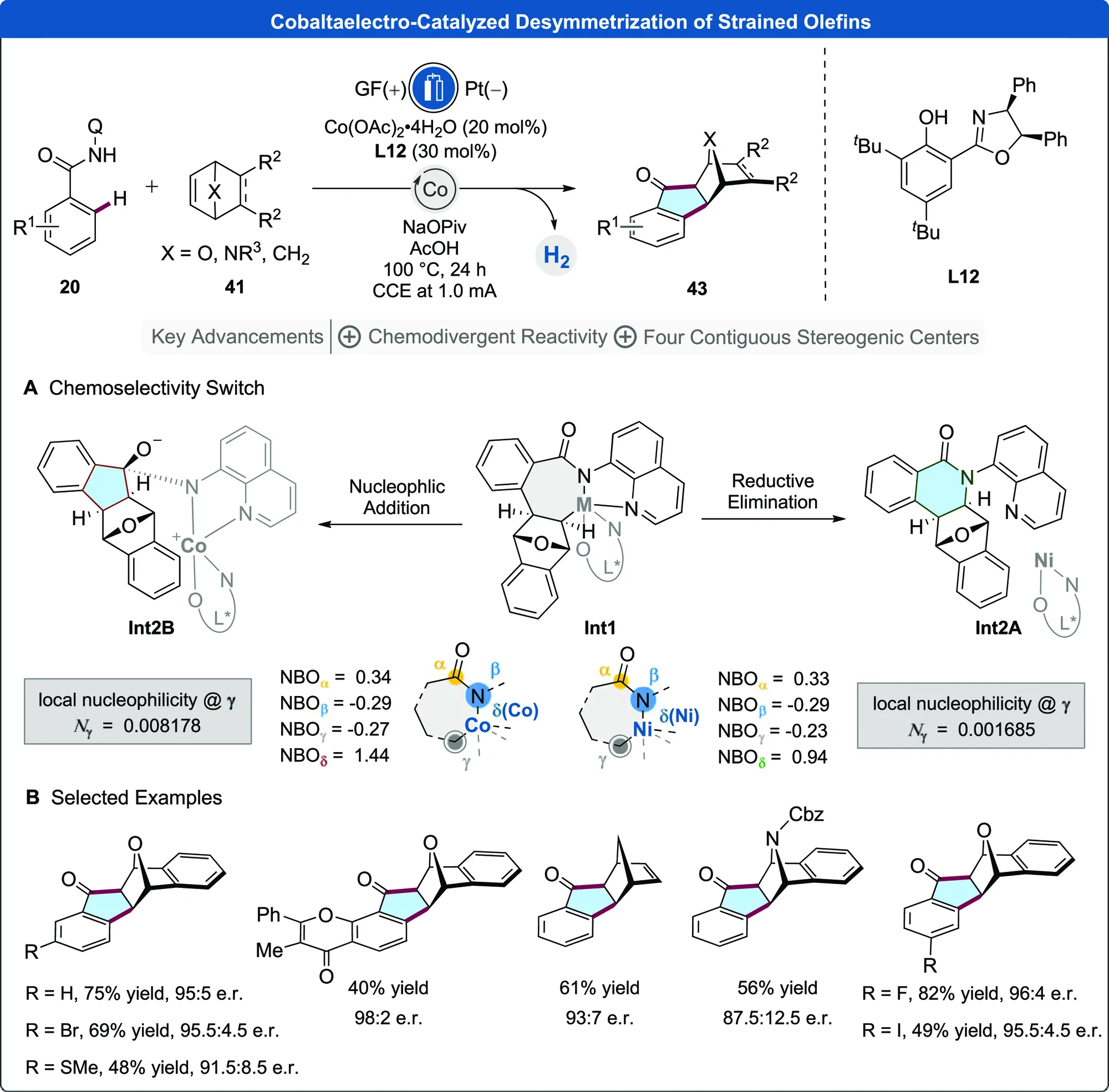
Enantioselective Cobaltaelectro-Catalyzed Carboacylation Toward Bicyclic Scaffolds **43**

## 4 Conclusion and Outlook

Molecular electrochemistry has been identified as a powerful platform to perform oxidative reactions under exceedingly mild conditions, circumventing stoichiometric chemical oxidants. The merger with transition metal-catalyzed C–H activation and particularly within enantioselective metallaelectro-catalysis, thus, granted access to a sustainable, versatile toolbox for the synthesis of complex chiral architectures. Initiated by palladaelectro-catalyzed enantioselective C–H activation reactions, rhodium- and ruthenium-catalyzed reactions were developed, showcasing the unique potential of metallaelectro-catalysis with chiral complexes, chiral transient directing groups, or chiral carboxylic acids. Substantial progress was achieved through the development of diverse electrochemical enantioselective transformations, employing environmentally benign and cost-efficient 3d transition metal catalysts, such as copper, cobalt, and nickel, with a chiral ligand for *in situ* association. The high efficacy and versatility of 3d metallaelectro-catalysis in the construction of diverse stereochemical elements demonstrated the enormous potential for organic synthesis, challenging noble metal catalysis. Overall, our efforts in metallaelectro-catalyzed C–H activation over the past decade provided means to assemble molecules with helical, point, axial, and planar chirality.

Despite these indisputable advances, enantioselective metallaelectro-catalyzed C–H activation represents a novel development with several unresolved challenges, requiring further innovations toward generality and broad applicability. While noble metal catalysis demonstrated the viability of weakly coordinating and synthetically useful functionalities, such as aldehydes or carboxylic acids, for directing diverse metallaelectro-catalyzed functionalizations, the lability of metal–ligand bonds and general high reactivity of 3d transition metals result thus far in transformations with rather strongly coordinating functional groups. Further, most transformations reported in this field rely on comparatively diluted conditions and moderate current densities. Thus, accessing generality across diverse substrate classes and achieving practicability on scale remain central challenges.

Accordingly, continuous ligand development and a deeper understanding of electrocatalytic mechanisms and stereoinduction will be essential. In particular, the implementation of data-driven approaches to identify optimal reaction conditions and design highly efficient chiral ligands is, thus, expected to further accelerate innovation within this rapidly evolving research area. Still, the predictive power of contemporary models is broadly limited by the quantity, quality, and diversity of the data because of a literature bias on positive results. Since informed ML approaches were shown to foster the development of specific transformations (*vide supra*), increasingly extrapolative models are expected to emerge, enabling vast acceleration of reaction discovery.

Given the increasing real-world importance of metallaelectro-catalysis, a broad wealth of further compelling advances is expected, diversifying applicability and practicality. On this note, photoelectrocatalysis was shown to foster mild and diverse redox processes through the interplay with light and, thus, holds enormous potential for selective catalysis.^147,148^ Notable recent developments include the implementation of scalable electrooxidative transformations with immense societal demand^149^ and metallaphotoelectro-catayzed late-stage functionalization,^150^ underlining this field’s excellent prospects.
